# Climate, Air Quality and Their Contribution to Cardiovascular Disease Morbidity and Mortality in Low- and Middle-Income Countries: A Systematic Review and Meta-Analysis

**DOI:** 10.5334/gh.1409

**Published:** 2025-03-27

**Authors:** Stephaney Gyaase, Solomon Nyame, Kerstin Klipstein-Grobusch, Kwaku Poku Asante, George S. Downward

**Affiliations:** 1 Kintampo Health Research Centre, Research and Development Division, Ghana Health Service, Kintampo, Ghana; 2 Julius Global Health, Department of Global Public Health and Bioethics, Julius Center for Health Sciences, University Medical Center Utrecht, Utrecht University, Utrecht, The Netherlands; 3 Division of Epidemiology and Biostatistics, School of Public Health, Faculty of Health Sciences, University of the Witwatersrand, Johannesburg, South Africa; 4 Institute for Risk Assessment Sciences, Utrecht University, The Netherlands

**Keywords:** cardiovascular disease morbidity, mortality, short-term, long-term, exposures

## Abstract

**Background:**

Increasing exposure to climatic features is strongly linked to various adverse health outcomes and mortality. While the link between these features and cardiovascular outcomes is well established, most studies are from high-income countries.

**Objectives:**

This review synthesizes evidence as well as research gaps on the relationship between climate indicators, household/ambient air pollution, and all-cause cardiovascular disease (CVD) morbidity and mortality in low- and middle-income countries (LMICs).

**Methods:**

Seven electronic databases were searched up to June 15, 2024. Articles were included if they focused on LMICs, addressed all-cause CVD morbidity and/or mortality, and studied climate or environmental exposures. Studies were selected using ASReview LAB, extracted and analyzed with random effect meta-analysis performed if sufficient articles were identified.

**Results & Conclusion:**

Out of 7,306 articles, 58 met the inclusion criteria: 26 on morbidity, 29 on mortality, and 3 on both. Exposures included PM_10_, PM_2.5_, NO_2_, SO_2_, BC, O_3_, CO, solid fuel usage, and temperature variation. Short-term exposure to PM_2.5_ was significantly associated with CVD morbidity (RR per 10 µg/m^3^ increase:1.006, 95% CI 1.003–1.009) and mortality (RR:1.007, 95% CI 1.002–1.012). Short-term exposure to NO_2_ and O_3_ also increased CVD mortality risk. Long-term exposure to PM_2.5_ elevated CVD morbidity (RR per 10 µg/m^3^ increase:1.131, 95% CI 1.057–1.210) and mortality (RR:1.092, 95% CI 1.030–1.159). High and low temperatures and long-term solid fuel use were linked to CVD deaths. The bulk of studies were from mainland China (72%), which may not accurately reflect the situation in other LMICs. Sub-Saharan Africa was particularly lacking, representing a major research gap.

## Introduction

The World Health Organization (WHO) estimates that cardiovascular diseases (CVD) are responsible for approximately 32% of all deaths, with more than 75% of these deaths occurring in low- and middle-income countries (LMICs). Within the regions of Sub-Saharan Africa, cardiovascular diseases are the most common cause of noncommunicable diseases (NCDs), with a regional burden that is anticipated to double by the year 2030 (1,2,3).

Climatic and environmental disruptions to health can be observed via multiple systems and pathways, affecting people throughout the life course. It is estimated that every year, air pollution is responsible for approximately 7 million premature deaths around the world, with cardiovascular diseases being among the top four diseases related to air and other environmental exposures (4,5,6,7,8). Several lines of evidence have demonstrated that increasing and ongoing exposure to climatic features such as heat waves, air pollution, and household air pollution can cause or are strongly related to myriad adverse long- and short-term health outcomes such as CVD, respiratory disorders, elevated blood pressure, malignancies, heart disease, and death (4,9,10,11,12,13,14,15,16,17,18,19,20,21,22,23,24).

However, the majority of these estimates are based on studies from high-income countries. As such, there is very little in terms of evidence synthesis from a primarily LMIC context. With the burden of CVD anticipated to double by the year 2030 (25), the limited health care infrastructure in LMIC settings and substantial differences between pollutant sources, extrapolations of evidence from high-income settings are of limited use. The purpose of this review, therefore, is to examine and synthesize the evidence related to key indicators of climate, household/ambient air pollution, and their association with all-cause CVD morbidity and mortality in LMICs. We will also evaluate relevant research gaps in this setting related to the link between climatic indicators and all-cause CVD morbidity and mortality in LMICs.

## Methods

### Search Strategy

The systematic review and meta-analysis protocol followed PRISMA guidelines and was registered with PROSPERO (registered ID: CRD42022373943). We searched seven electronic databases, including PubMed, Embase, SCOPUS, LILACS, AIM, Web of Science, and Global Health. Specific search terms for each database are included in the appendix.

### Eligibility criteria

Studies were eligible for inclusion in the current study if they contained information related to climate and environmental exposures and their effect on all-cause cardiovascular disease morbidity and mortality amongst adults (i.e., 18 years and older) in low- and middle-income countries. A wide range of reporting all-cause CVD morbidity/mortality was allowed, including physician diagnosis, self-report, national disease surveillance database, hospital records, verbal autopsies, and death certificates. The definition of LMIC was based on the World Bank definition at the time of the literature search. Studies that did not primarily examine environmental conditions, examine pediatric populations, have a high-income-country focus, or were restricted to specific cardiovascular conditions (e.g., hypertension alone) were not included.

There was no restriction on the publication date. The search was initially performed on November 20, 2023, and updated on June 15, 2024.

### Article Selection and data extraction

All articles identified by the initial searches were exported to Rayyan (26) for deduplication. Following this, the remaining articles were imported to ASReview lab version 1.1.1 (27) for title and abstract screening. ASReview is an artificial intelligence tool that utilizes a TF-IDF, Naïve Bayes, mixed sampling model to rank titles and abstracts based on their probability of being relevant. This probability is initially based on positive examples provided by the reviewer and subsequently refined following inclusion or exclusion decisions. Titles that are less likely to qualify for the current review are ranked lower, with the ranking being dynamically updated following each decision. A stopping rule based on a data-driven strategy was employed by two reviewers (SG and GD) and set at 250 consecutive irrelevant articles. Any disagreement about the articles selected was discussed, and an agreement was reached. Articles identified through title/abstract screening subsequently underwent full-text screening. Articles retained following full-text screening underwent data extraction for information on author, title, publication year, study location, study period, study design, outcome type, mean/median exposure, comorbidities controlled for, total population, and type of pollution or climatic factor exposure studied.

### Risk of bias assessment

The quality and risk of bias of the identified studies were evaluated by two reviewers (SG and GD) using the study Quality Assessment Tool of the National Health Institute/National Heart, Lung and Blood Institute (NHI/NLBI) (28). The tool has a rating of good, fair, and poor based on fourteen criteria assessments. Ratings of poor quality are associated with high risk of bias, with fair and good being associated with medium and low risks of bias, respectively.

### Statistical Analysis/Meta-analysis

Studies were pooled and examined based on whether they examined either short (e.g., time-series analysis) or long (e.g., cohort) term exposures. In addition, studies examining similar environmental components and designs were pooled via random-effect meta-analysis. When examining short-term exposures, the longest combined (i.e., cumulative or pooled) lag effect (e.g., pooled lag over days 0–7) reported was retained for analysis. This decision was made to avoid selecting only lag estimates with positive findings. Effect estimates (Risk Ratio (RR)/Odds Ratio (OR), Hazard Ratio (HR), percentages) and 95% confidence intervals (95% CI) or sufficient information included for estimates calculation were extracted and configured to indicate the impact per specific increment in exposure. Exposures to PM_10_, PM_2.5_, NO_2_, SO_2_, BC, and O_3_ were pooled for a 10 µg/m^3^ increment, and CO exposure was pooled for a one part per million (ppm) increment. Exposure to solid fuel was based on solid fuel versus clean fuel. Studies reporting percentage change were converted to RR using the below formula:

\[RR{\mathrm{}}\;{\mathrm{ = }}\;\frac{{{\mathrm{Percentage}}\;{\mathrm{change}}}}{{100}}\;{\mathrm{ + }}\;1\]

A forest plot was used to visualize the summaries of the included studies. Both visual inspection of the forest plot and I^2^ statistics were used to assess the degree of heterogeneity of the true effect. Results of I^2^ < 30%, 30%–50%, and >50% were interpreted to indicate no, moderate, and substantial heterogeneity, respectively. Begg’s and Egger’s tests were used to assess publication bias and small study effects for meta-analysis involving five or more studies (29). As the association between temperature and morbidity/mortality tends to follow a U or J shape, meta-analysis was not performed. Instead, we described these effects at high and low temperatures. All analyses were conducted using R version 4.3.1 (30), and a two-sided P < 0.05 was deemed statistically significant.

## Results

**Figure 1:**
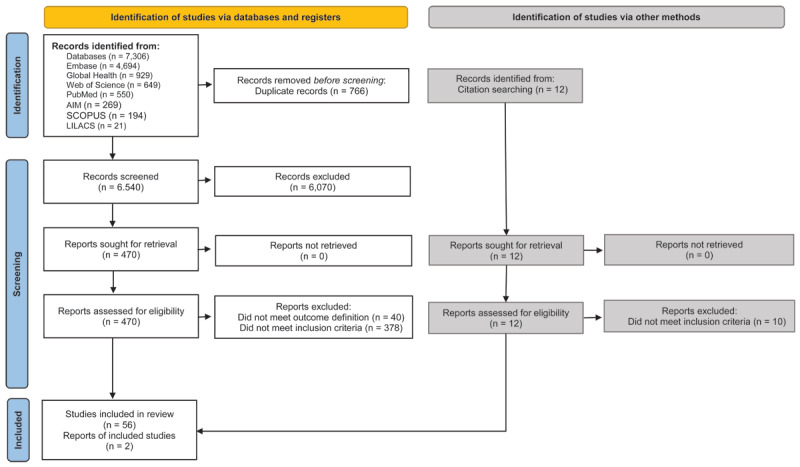
Description of the articles selection processes.

In total, the search returned 7,306 articles across the seven databases. After duplicate removal, 6,540 articles remained. After title and abstract screening, 58 papers were retained for data extraction and analysis—26 of which examined morbidity, 29 examined mortality, and 3 examined both. For details regarding article selection, see Figure 1. Nine different exposure parameters were examined (PM_10_, PM_2.5_, SO_2_, NO_2_, O_3_, temperature variation, CO_2_, Black Carbon, and solid fuel), with the number examined per paper ranging from one to six (the average examined was approximately two). Single-exposure models were most used, with a smaller number of articles utilizing multi-exposure models in secondary analysis. We therefore primarily focused on the findings of single exposure models. A variety of methods of reporting CVD morbidity/mortality were used, including physician diagnosis, self-report, national disease surveillance database, verbal autopsy, death certificate, and hospital records. In terms of study location, the majority (n = 42, 72%) of studies were conducted in Mainland China. Regarding other regions, 4 studies were conducted in Iran, 3 each in Thailand and South Africa, 2 each in Vietnam and Bangladesh, and 1 in Brazil. Only one study was conducted in multiple countries, thus; China, India, South Africa, and Tanzania. Most (n = 53) papers had a moderate to low risk of bias, with 51 papers being determined as a ‘fair’ quality and 5 as ‘good’ (Table 1).

**Table 1:** Description of included articles on CVD morbidity/mortality and environmental factors.


AUTHOR & YEAR	LOCATION	STUDY DESIGN	MAIN EXPOSURE(S)	OUTCOME AND DEFINITION	MAIN FINDINGS	STUDY QUALITY

Buadong et al., 2009 (33)	Bangkok, Thailand	Time-series	PM_10_, O_3_	Morbidity – daily hospital visits	There was no significant association for either PM_10_ or O_3_ on CVD morbidity in the 3-day cumulative lag model.	Fair

Dong et al., 2013 (56)	Liaoning Province, China	Cross-sectional	PM_10_, SO_2_, NO_2_, O_3_	Morbidity – Positive response from questionnaire	No significant association was found between any of the air pollutants and CVD morbidity	Fair

Tong et al., 2014 (36)	Tianjin Municipality, China	Time-series	PM_10_, SO_2_, NO_2_	Morbidity – Database	A 10 µg/m^3^ increase in the 2-day average concentration of PM_10_ and SO_2_ were associated with a 0.19% (0.08–0.31) and 0.43% (0.03–0.84) increase in CVD morbidity respectively. No significant association was found for NO_2_.	Fair

Giang et al., 2014 (31)	Thai Nguyen, Vietnam	Time-series	Temperature	Morbidity – Hospital admission	Over a 0–30-day lag period, there was a 12% (1%–25%) increase in CVD hospital admissions per 1 degree below the temperature threshold. A positive, yet non-significant association was observed for increased temperature.	Fair

Su et al, 2016 (34)	Haidian District, Beijing, China	Time-series	PM_10_, PM_2.5_, SO_2_, NO_2_	Morbidity – Medical records of emergency visits	In the 0–7-day cumulative lag model, no significant association between PM_2.5_, PM_10_, SO_2_, or NO_2_, and CVD morbidity was observed.	Fair

de Freitas et al., 2016 (35)	Victoria, Brazil	Time-series	PM_10_, O_3_, SO_2_,	Morbidity – Hospital records	In the 0–5-day cumulative lag model, CVD events increased by 2.11% (1.06–3.18) per 10 µg/m^3^ increase in O_3_. No significant association was observed for PM_10_ and SO_2_.	Poor

Phung et al., 2016 (39)	Vietnam	Time-series	PM_10_, SO_2_, NO_2_, O_3_	Morbidity – Hospital admission	In the lag-3 model, neither PM_10_, NO_2_, SO_2_ or O_3_ had a statistically significant association with CVD morbidity.	Fair

Ma et al., 2017 (42)	Beijing, China	Time-series	PM_10_, SO_2_, NO_2_	Morbidity – Hospital admission	For a 10 µg/m^3^ increase in NO_2_, ER cardiovascular admission increased by 1.4% (RR:0.986; 95%CI:0.976–0.996) in the 0–6-day cumulative lag model. There was no association between CVD admission and PM_10_ or SO_2_.	Fair

Liu et al., 2018 (46)	Mainland China	Case crossover	CO	Morbidity – Health database	A 1 mg/m^3^ increase in the same day CO was associated with a 4.39% (4.07–4.70) increase in CVD.	Fair

Li et al., 2018 (47)	Beijing, China	Case crossover	CO	Morbidity – Health database	A 1 mg/m^3^ increase in the 2-day moving average of CO was associated with a 2.8% (2.2–3.3) increase in daily hospital CVD admissions.	Fair

Phosri et al., 2019 (38)	Bangkok, Thailand	Time-series	SO_2_, NO_2_, O_3_, CO	Morbidity – Daily hospital admission	A 10 µg/m^3^ increase in PM_10_, SO_2_, and NO_2_ corresponded to 0.6% (0.10–1.00), 5.3% (2.42–8.21), and 0.6% (0.06–1.09) increases in total CVD admission in the 0–4-day cumulative lag models, respectively. A 1 mg/m^3^ increase in CO increased CVD admission by 4.2% (1.35–7.26). No significant association with O_3_ was observed.	Fair

Yao et al., 2019 (86)	Yichang Province, China	Time-series	PM_10_, PM_2.5_	Morbidity-Daily inpatient records	There was no statistically significant association between PM_10_ or PM_2.5_ and CVD admission in the lag 7 model.	Fair

Amsalu et al., 2019 (32)	Beijing, China	Time-series	PM_2.5_	Morbidity – Daily hospital admission	In the 0–3-day lag model, a 10 µg/m^3^ increase in PM_2.5_ was associated with a 0.7% (0.4–0.9) increase in CVD hospital admissions.	Fair

Cheng et al., 2019 (48)	Lanzhou city, China	Time-series	CO	Morbidity – Daily CVD hospitalization	In the lag 0–4 model, a 1 mg/m^3^ increase in CO was associated with an 11% increase (95%CI: 3%–20%) in CVD hospitalization.	Fair

Khan et al., 2019 (45)	Dhaka, Bangladesh	Case crossover	PM_2.5_	Morbidity – Emergency room visit	An IQR increase (103 µg/m) of PM_2.5_ corresponded to a 15% increase (1–30) in CVD emergency room visits in the 3–5-day lag model.	Fair

Phosri et al., 2020 (43)	Bangkok, Thailand	Time-series	Temperature	Morbidity – Daily hospital admission	In the 0–21 lag models, an “extremely high” diurnal temperature range (11.6°C) was associated with a 20.6% (0.2–45.2) increase in CVD hospital admissions.	Fair

Rahman et al., 2022 (40)	Dhaka, Bangladesh	Time-series	Temperature	Morbidity – Count of CVD from Database	There was no association between a 1°C increase in temperature variability and ED visits for cardiovascular disease.	Fair

Karbakhsh et al., 2022 (44)	Iran	Case crossover	PM_10_, PM_2.5_, PM_coarse_	Morbidity – CVD admitted	An IQR increase in PM_coarse_ (IQR: 55 µg/m^3^) and PM_10_ (IQR: 71 µg/m^3^) was associated with an increase in CVD admission (OR:1.02; 95% CI: 1.00–1.05 and 1.02; 95% CI:1.01–1.04) respectively in the lag 0–1–2 model. No significant effect was observed for PM_2.5._	Fair

Makunyane et al., 2023 (37)	Cape Town, South Africa	Time-series	Temperature	Morbidity – Daily counts of hospital admission	An IQR (6.4°C) increase in temperature variability of TV was associated with a 2.61% (1.15–4.08) increase in hospital admissions.	Fair

Ji et al., 2021 (49)	Mainland China	Cohort	Solid fuel	Morbidity – Response from questionnaire	Individuals using solid fuels at baseline had a higher risk of non-fatal CVD event than those using clean fuels (HR:1.18; 95% CI:1.05–1.32).	Fair

Liu et al., 2021 (50)	Mainland China	Cohort	PM_2.5_	Morbidity – Based on Disease classification	An IQR increase in PM_2.5_ (27.9 µg/m^3^) increased the risk of CVD morbidity (HR:1.291, 95% CI: 1.147–1.54).	Fair

Mai et al., 2032 (51)	Mainland China	Cohort	PM_2.5_	Morbidity – Response from questionnaire	A 10 µg/m^3^ increase in PM_2.5_ was associated with an increased risk of CVD morbidity (OR:1.18 95% CI: 1.12–1.26).	Fair

Wen et al., 2023 (52)	Mainland China	Cohort	Solid fuel	Morbidity – Self Assessment	Treatment effect of cardiovascular disease after implementation of coal-to-gas/electricity project was not statistically significant.	Fair

Wang et al., 2023 (53)	Mainland China	Cohort	NO_2_	Morbidity – Questionnaire	A 10 µg/m^3^ increase in NO_2_ resulted in an elevated risk of CVD morbidity (HR:1.558 95% CI: 1.477–1.642).	Fair

Liu et al., 2023 (54)	Mainland China	Cohort	Solid fuel	Morbidity – Response from questionnaire	The use of solid fuel for cooking and heating versus clean fuel increased the risk of nonfatal CVD incident by 43.0% [HR:1.43 (1.07–1.92)].	Fair

Zhu et al., 2024 (55)	Mainland China,	Cohort	O_3_	Morbidity – Questionnaire	A 10 µg/m^3^ increase in long-term O_3_ exposure was positively associated with incident of CVD (HR:1.078 95% CI: 1.050–1.106).	Fair

Xia et al., 2023 (85)	Mainland China	Cohort	PM_2.5_	Morbidity & Mortality – Questionnaire	A 10 µg/m^3^ increase in PM_2.5_ was positively associated with total CVD morbidity (HR:1.12, 95% CI: 1.11–1.14) and CVD mortality (HR:1.12 95% CI: 1.08–1.15).	Good

Liang et al., 2020 (84)	Mainland China	Cohort	PM_2.5_	Morbidity & Mortality – Extracted from questionnaire	A 10 µg/m^3^ increase in PM_2.5_ gave HRs for CVD incidence and mortality of 1.25(1.22–1.28) and 1.16 (1.12–1.21), respectively.	Good

Jalali et al., 2021 (23)	Isfahan, Iran	Cohort	PM_2.5_	Morbidity & Mortality – Questionnaire & health records	The risk of CVD event increased by 2.6% (OR:1.026, 95% CI:1.016–1.036) for a 10 µg/m^3^ increase in PM_2.5_. No significant association was observed between PM_2.5_ and CVD mortality.	Fair

Zhang et al., 2006 (57)	Shanghai, China	Time-series	O_3_	Mortality – Database	An increase of 10 µg/m^3^ in the 4-day O_3_ average corresponded to a 0.9% increase (95% CI: 0.5–1.4) in total cardiovascular mortality.	Fair

Tam et al., 2010 (58)	Hong Kong Administrative Region	Time-series	Temperature	Mortality – Database	In the 0–3 lag model, a 1°C increase in diurnal temperature range resulted in a 1.7% increase in cardiovascular mortality (RR:1.017, 95% CI: 1.003–1.033)	Poor

Yang et al., 2012 (87)	Suzhou Province, China	Time-series	O_3_	Mortality – Database	An IQR increase in the 24-hour average concentration of O_3_ (33.3 µg/m^3^) was associated with a 3.33% (95% CI: 0.50–6.16) increase in CVD mortality.	Fair

Chen et al., 2012 (60)	Mainland China	Time series	SO_2_	Mortality – Database	A 10 µg/m^3^ increase in the 2-day moving average of SO_2_ was associated with a 0.83% increase in cardiovascular mortality (95% PI:0.47–1.19).	Fair

Wichmann & Voyi, 2012 (76)	South Africa	Case crossover	PM_10_, SO_2_, NO_2_,	Mortality – Database	There was a 3.4% (0.3–6.6) and 2.6% (0.1–5.2) increase in cardiovascular mortality per IQR increase in NO_2_ (IQR: 12 µ/m^3^) and SO_2_ (IQR: 8 µg/m^3^), respectively. No significant effect of PM_10_ was observed.	Fair

Fuhai Geng et al., 2013 (61)	Shanghai, China	Time-series	BC & PM_2.5_	Mortality – Database	An IQR increase in the mean daily concentrations of BC (IQR: 2.7 µg/m^3^) and PM_2.5_ (IQR: 41.8 µg/m^3^) corresponded to a 3.2% (0.6–5.7) and 3.3% (0.4–6.1) increase in total cardiovascular mortality, respectively.	Fair

Wang et al., 2014 (62)	Suzhou Province, China	Time-series	Temperature	Mortality – Database	In the 0–28 lag model, extreme cold (1^st^ centile: –0.3°C) and hot (99^th^ centile: 32.6°C) temperatures were positively associated with cardiovascular mortality with RRs of 2.67 (1.64–4.33) and 1.62 (1.21–2.17), respectively.	Fair

Han et al., 2017 (63)	Jinan Province, China	Time-series	Temperature	Mortality – Database	Cold spells (3 consecutive days below –3.8°C) and heat waves (3 consecutive days above 29°C) were associated with CVD mortality RRs of 1.06 (1.03–1.10) and 1.03 (1.00–1.06), respectively	Fair

Chen et al., 2018 (65)	Mainland China	Time-series	NO_2_	Mortality – Database	A 10 µg/m^3^ increase in the 2-day average concentration of NO_2_ would increase total cardiovascular mortality by 0.9% (0.7–1.2)	Fair

Chen et al., 2018 (64)	Mainland China	Time-series	PM_2.5_	Mortality – Database	In the 0–2 lag model, no significant association between PM_2.5_ and cardiovascular mortality was observed.	Fair

Liu et al., 2018 (66)	Mainland China	Time-series	CO	Mortality – Database	In the 0–1 lag model, a 1 mg/ m^3^ increase in CO was associated with a 1.12% (PI:0.42–1.83) increase in cardiovascular mortality	Fair

Wu et al., 2018 (67)	Guangzhou Province, China	Time-series	PM_2.5_, PM_10_ & PM_10-2.5_	Mortality – Database	In the lag 06 model, a 10 µg/m^3^ increase in PM_2.5_, PM coarse, and PM_10_ was associated with an excess risk for CVD mortality of 1.15% (95% CI: 0.68, 1.62), 1.64% (95% CI: 0.86, 2.43), and 0.82% (95% CI: 0.49, 1.14), respectively.	Fair

Zhang et al., 2019 (41)	Jiangsu Province, China	Time-series	O_3_	Mortality – Database	In the lag 0–3 model, a 10 µg/m^3^ increase in O_3_ was associated with a 0.983% (0.588–1.3770) increase in CVD-related death.	Fair

Liu et al., 2019 (68)	Shenyang Province, China	Time-series	PM_10_, PM_2.5_, SO_2_, NO_2_, O_3_, CO	Mortality – Death registry	In the lag 05 model, 10 µg/m^3^ increases in PM_2.5_, PM_10_, SO_2_, and NO_2_ were associated with RRs for CVD mortality of 1.004 (1.001, 1.008), 1.003 (1.001, 1.006), 1.005 (1.001, 1.009), and 1.016 (1.005, 1.028), respectively. A 1 mg/m^3^ increase in CO was associated with an RR of 1.066 (1.025, 1.108). No significant association was observed for O_3._	Fair

Duan et al., 2019 (69)	Shenzhen Province, China	Time-series	NO_2_	Mortality – Database	In the lag 0–5-day model, a 10 µg/m^3^ increase in NO_2_ was associated with a 3.41% (1.55–5.30) increase in cardiovascular mortality.	Fair

Iranpour et al., 2020 (70)	Ahvaz, Iran	Time-series	Temperature	Mortality – Database	In the 0–28-day lag model, no association between heat above the 99^th^ centile (41.2°C) or below the 1^st^ centile (9.3°C), and CVD mortality was observed.	Fair

Khosravi et al., 2020 (71)	Mashhad, Iran	Time-series	PM_10_, PM_2.5_, NO_2_, O_3_, CO	Mortality – Database	None of the five pollutants assessed were associated with cardiovascular mortality.	Fair

Zhou et al., 2021 (72)	Taiyuan Province, China	Time-series	PM_10_, PM_2.5_	Mortality – Database	In the 0–30 lag model, a 10 µg/m^3^ increase in PM_2.5_ and PM_10_ was associated with a 3.10% (0.86–5.38) and 1.61% (0.69–2.54) increase in cardiovascular mortality.	Fair

Li et al., 2021 (73)	Guangzhou Province, China	Time-series	O_3_	Mortality – Registry	In the 0–3 lag model, a 10 µg/m^3^ increase in O_3_ was associated with a 0.59% (0.30–0.88) increase in CVD mortality.	Fair

Olutola et al., 2023 (75)	South Africa	Case crossover	PM_10_, SO_2_, NO_2_,	Mortality – Database	In the 0–6-day lag model, none of the examined pollutants were associated with increased CVD mortality.	Fair

Xia et al., 2023 (74)	Chengdu, China	Time-series	Temperature	Mortality – Database	In the 0–14-day lag model, extreme heat (99^th^ centile, >29 °C) and extreme cold (1^st^ centile, < 3°C) were both associated with increased CVD mortality, with RRs of 1.28 (1.14–1.43) and 1.45 (1.24–1.68), respectively.	Fair

Cao et al., 2011 (59)	Mainland China	Cohort	SO_2_, TSP, NO_X_	Mortality – Hospital records	A 10 µg/m^3^ increase in TSP, SO_2_, and NOx corresponded to 0.9% (95% CI: 0.3, 1.5), 3.2% (95% CI: 2.3, 4.0), and 2.3% (95% CI:0.6, 4.1) increases in cardiovascular mortality, respectively.	Fair

Wong et al., 2015 (77)	Hong Kong, Administrative Region	Cohort	PM_2.5_	Mortality – Death registry	A 10 µg/m^3^ increase in PM_2.5_ exposure was associated with a 22% increase in cardiovascular mortality [HR:1.22 (1.08–1.39)].	Fair

Yu et al., 2018 (78)	Mainland China	Cohort	Solid fuel	Mortality – Questionnaire	Solid fuel use for cooking or heating was significantly associated with higher risk of cardiovascular mortality [HR:1.20 (1.02–1.41)] and [HR:1.29 (1.06–1.55)], respectively.	Fair

Yang et al., 2018 (79)	Mainland China	Cohort	PM_2.5_, NO_2_ & BC	Mortality – Database	An IQR increase in PM_2.5_ (5.5 µg/m^3^) or BC (9.6 µg/m^3^) was associated with increased HRs for CVD mortality (1.06 [1.02–1.10] and 1.07 [1.02–1.11], respectively. No significant association was observed for NO_2._	Fair

Arku et al., 2020 (80)	China, India, South Africa and Tanzania	Cohort	Kerosene	Mortality – Hospital records, Death certificate and Verbal autopsies	Household cooking primary with kerosene had a 34% [HR:1.34 (1.08–1.66)] increase in major cardiovascular disease mortality.	Fair

Liang et al., 2022 (81)	Mainland China	Cohort	PM_2.5_	Mortality – Death registry	A 10 µg/m^3^ increase in PM_2.5_ was associated with a HR for cardiovascular mortality of 1.02 (1.00–1.05).	Good

Liu et al., 2022 (82)	Yinzhou Province, China	Cohort	O_3_	Mortality – Death registry	A 10 µg/m^3^ increase in long-term annual average of O_3_ increased cardiovascular mortality by approximately 22% [HR:1.22 (1.12–1.33)].	Good

Niu et al., 2022 (83)	Mainland China	Cohort	O_3_	Mortality – Death registry	A 10 µg/m^3^ increase in O_3_ was associated with an elevated risk of cardiovascular mortality [HR:1.093 (1.046–1.142)].	Good


The relationship between environmental exposure(s) and CVD morbidity/mortality was examined either through the relationship between short-term changes in exposure and acute events or through long-term exposures and CVD disease. Short-term exposures were typically examined through a lag of up to 7 days, except for studies examining temperature, where lags of up to 28 days were also observed. Of the twenty-six articles examining cardiovascular morbidity, 17 assessed the effect of short-term exposures (of which 13 used time series (31,32,33,34,35,36,37,38,39,40,41,42,43), and 4 used a case-crossover design (44,45,46,47)). Among the 9 studies examining long-term exposures, 8 utilized cohort studies (48,49,50,51,52,53,54,55), and 1 used a cross-sectional approach (56). Of the 29 articles examining cardiovascular mortality, 21 examined short-term effects (of which 19 used a time-series design (41,57,58,59,60,61,62,63,64,65,66,67,68,69,70,71,72,73,74) and 2 used a case-crossover design (75,76), with the remaining 8 articles examining long-term effects by use of a cohort study design (59,77,78,79,80,81,82,83). The 3 articles assessing the effect on both morbidity and mortality all examined long-term effects by use of a cohort design (23,84,85).

### 1.1: The effect of short-term exposures on cardiovascular morbidity and mortality

#### 1.1.1: Exposure to PM_10_

The effects of short-term exposure to PM_10_ on CVD morbidity and mortality were reported by nine and six articles, respectively, typically showing a combination of increased or null likelihoods of disease or mortality. Of the nine morbidity articles, eight (33,34,35,36,38,39,42,86) utilized a time series design, with the remaining article utilizing a case-crossover design (44). The time-series articles were retained for meta-analysis. The case-crossover study reported that for a 10 µg/m^3^ increase in PM_10_, the risk of CVD morbidity increases by 0.6% (95% CI: 0.4–0.8) (44). In the meta-analysis, a 10 µg/m^3^ increase of PM_10_ was found to increase short-term CVD morbidity by 0.1% (i.e., an RR of 1.001) with a 95% confidence interval (CI) of 0.0% to 0.3%. There was no evidence of publication bias for morbidity outcomes (Begg’s test, p = 0.4885, and Egger’s test, p = 0.3988).

With regards to the six articles examining short-term exposure to PM_10_ and CVD mortality, four utilized a time series design (67,68,71,72) and were included in the meta-analysis. The remaining two (33,76) utilized a case-crossover design, neither of which displayed significant results in their maximal lag models. The pooled meta-analysis found that, for a 10 µg/m^3^ increase in PM_10_, the risk of CVD-related death increases by 0.7% (RR: 1.007 95% CI: 1.000–1.014)]. Publication bias was not assessed for mortality due to the relatively small number of papers. Further details are presented in Supplementary Figure A1.

#### 1.1.2: Exposure to PM_2.5_

Short-term exposure to PM_2.5_ and its effects on CVD morbidity were assessed by four articles (32,34,44,86), and its effects on mortality were assessed by six (61,64,67,68,71,72), with studies tending to show positive associations, albeit with a combination of significant and non-significant effects. Of the four morbidity studies, three (32,34,86) represented time-series design and were included in the meta-analysis (the remaining case-crossover study did not identify a significant relationship between PM_2.5_ and CVD morbidity). The pooled meta-analysis found that a 10 µg/m^3^increase in PM_2.5_ was associated with a 0.6% increase in CVD morbidity (RR: 1.006, 95% CI: 1.003–1.009). Due to the small number of articles examining morbidity, a test of publication bias was not performed, and no heterogeneity was observed.

**Figure 2:**
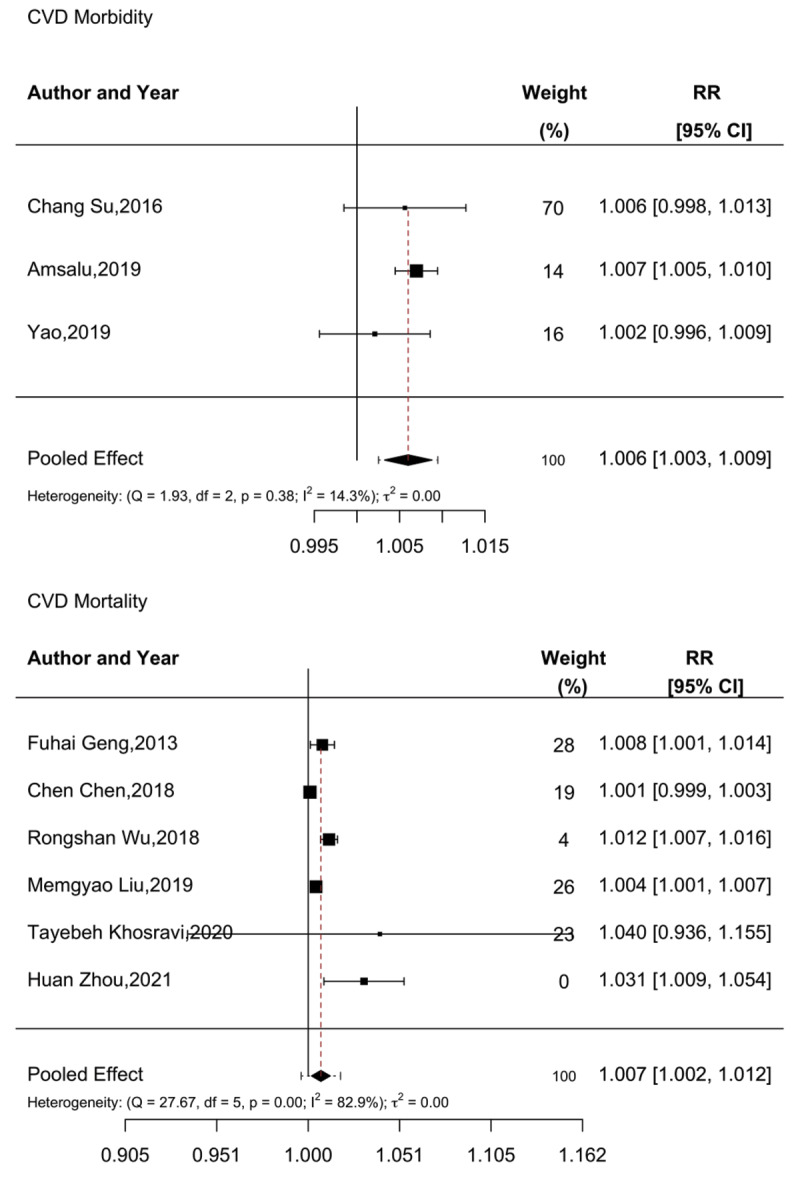
Meta-analysis of short-term PM_2.5_ exposure and CVD morbidity and mortality.

For the effects of short-term PM_2.5_ exposure on CVD-related mortality, all six studies utilized a time-series design and were included in the meta-analysis. The overall pooled results indicated that a 10 µg/m^3^increase in PM_2.5_ corresponded to a 0.7% increase in CVD mortality (RR: 1.007, 95% CI: 1.002–1.012). There was some evidence of publication bias (Begg’s test, p = 0.015; Egger’s test, p = 0.7194), and substantial heterogeneity was observed. Further details are presented in Figure 2.

#### 1.1.3: Exposure to NO_2_

The short-term effects of NO_2_ exposure on CVD morbidity and mortality were evaluated by five and six studies, respectively. Overall, the association between NO_2_ and CVD morbidity was mixed, with a more positive association observed with mortality. All five studies examining CVD morbidity were of time-series design and thus included in the meta-analysis (34,36,38,39,42). In pooled meta-analysis, no significant association between NO_2_ exposure and CVD morbidity was observed [RR: 1.00 (95% CI: 0.991–1.008)]. No publication bias was observed (Begg’s test, p = 0.5109, and Egger’s test, p = 0.2333).

**Figure 3:**
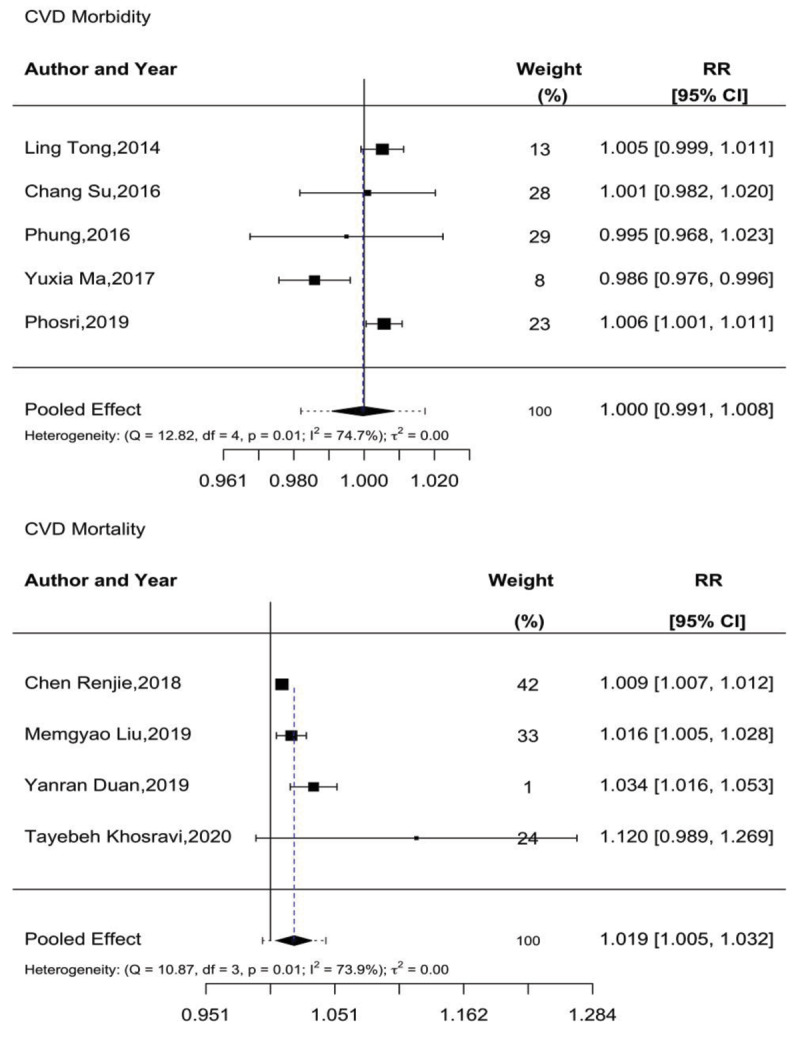
Meta-analysis of short-term NO_2_ exposure and CVD morbidity and mortality.

When examining the effect of NO_2_ on CVD mortality, four out of the six studies utilized a time-series design and were included in the meta-analysis. The remaining two articles, utilizing a case-crossover design, showed no association between NO_2_ and mortality in their maximal lag models (75,76). Meta-analysis found that a 10 µg/m^3^ increase in NO_2_ resulted in a 1.9% [RR: 1.019 (95% CI: 1.005–1.032)] increase in CVD-related deaths. Some evidence of publication bias was observed (Begg’s test, p < 0.0001, and Egger’s test, p = 0.0833). Further details are presented in Figure 3.

#### 1.1.4: Exposure to O_3_

The effects of O_3_ exposure on CVD morbidity and mortality were assessed by four and six articles, respectively, with limited evidence being observed for an association between O_3_ and morbidity, although there was more consistent evidence for an increased association with CVD mortality. All four articles examining morbidity utilized a time-series design and were included in the meta-analysis (33,35,38,39), which found no overall association between a 10 µg/m^3^ increase in O_3_ exposure and CVD morbidity (RR: 1.004, 95% CI: 0.995–1.014) (35).

**Figure 4:**
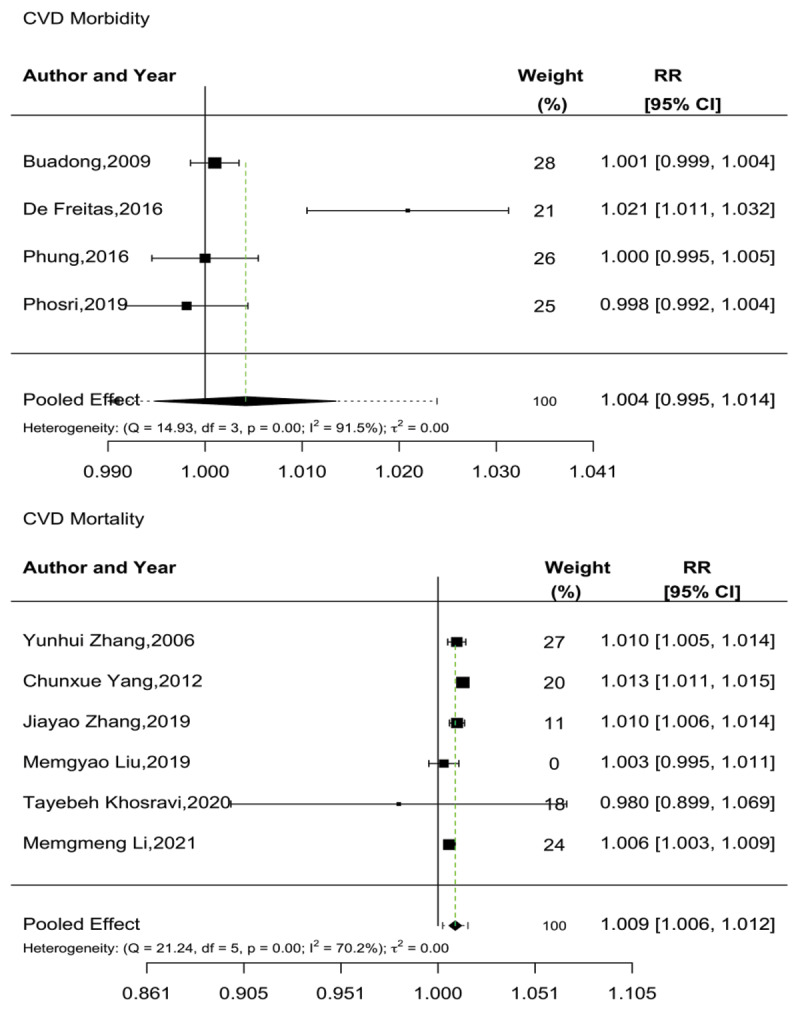
Meta-analysis of short-term O_3_ exposure and CVD morbidity and mortality.

For CVD mortality, all six articles utilized a time-series design and were thus included in the meta-analysis (41,57,68,71,73,87), which indicated that a 10 µg/m^3^ increase in O_3_ exposure was associated with a 0.9% increase in CVD-related mortality [RR: 1.009 (95% CI: 1.006–1.012)]. No evidence of publication bias was found in the mortality outcomes (Begg’s test, p = 0.1949, and Egger’s test, p = 1.7194). Further details are presented in Figure 4.

#### 1.1.5: Exposure to SO_2_

Four and six articles examined the short-term effects of SO_2_ on CVD morbidity and mortality, respectively. All six morbidity articles incorporated a time-series design and were included in the meta-analysis (34,35,36,38,39,42), which did not show a significant pooled association (RR: 1.006 95% CI: 0.993–1.020). No evidence of publication bias was observed (Begg’s test, p = 0.0492, and Egger’s test, p = 0.4694). Details of the meta-analysis are found in Supplementary Figure A2.

Meta-analysis was not performed to examine the pooled effect of SO_2_ on CVD mortality as two papers utilized time-series design and the other two case-crossover. However, three of the four articles reported a positive and significant association (60,68,76), with effect sizes ranging from 0.8% (95% CI: 0.47–1.19) to 3.3% (95% CI: 0.06–7.9) per 10 µg/m^3^ increment, with the fourth reporting no significant association (75).

#### 1.1.6: Exposure to CO

Two articles examined the impact of CO on CVD morbidity, and three examined mortality. Due to the limited number of articles, a meta-analysis was not performed. Both articles examining morbidity reported a positive association with a 1 ppm increase in CO exposure increasing morbidity by 4.2% (95% CI: 1.35–7.26%) and 11% (3–20%). Two of the three articles examining the effect of CO on mortality reported a positive effect (66,68), with results ranging from 1.1% (0.42–1.83%) to 6.5% (2.5–10.8%). The final article observed no significant association between CO and CVD mortality.

#### 1.1.7: Temperature exposure

Four and five articles assessed the effect of temperature variation on CVD morbidity and mortality, respectively, with effects observed at temperatures that were both higher and lower than normal temperatures. Out of the four articles examining temperature variation on CVD morbidity, two reported a significant association between higher-than-normal temperatures and CVD morbidity (37,43). One reported that lower-than-normal temperatures increased CVD morbidity (with no significant effect for hot temperatures reported) (31). The final article reported no relationship between temperature variation and CVD morbidity (40).

Among the five articles examining temperature and CVD mortality, three reported significantly increased mortality associated with both abnormally high and low temperatures. One article reported an association only for increased temperature, and one reported no association between temperature and mortality in its maximally lag-adjusted model.

#### 1.1.8: Other components

One article examined the impact of Black Carbon exposure on CVD mortality, reporting that an IQR increase (2.7 µg/m^3^) in BC was associated with a 3.2% (95% CI: 0.6–5.7%) increase in CVD mortality. Two articles examined PMcoarse, one examining morbidity and the other mortality. Both articles reported a positive association between exposure and morbidity/mortality.

### 1.2: The effect of long-term exposures on cardiovascular morbidity and mortality

#### 1.2.1: Exposure to PM_10_

Only one article assessed the long-term effect of PM_10_ on CVD morbidity, reporting no effect in cross-sectional analysis (56). The impact of PM_10_ on CVD mortality was not assessed.

#### 1.2.2: Exposure to PM_2.5_

CVD morbidity and mortality in relation to long-term PM_2.5_ exposure was assessed by five and six articles, respectively, consistently showing increased associations between PM_2.5_ and CVD morbidity/mortality. All five articles evaluating morbidity utilized a cohort design and were included in the meta-analysis (23,50,51,84,85), finding that a 10 µg/m^3^ increase in PM_2.5_ exposure increased CVD morbidity by approximately 13.1% [RR: 1.131 (95% CI: 1.057–1.210)]. No evidence of publication bias was observed (Begg’s test, p = 0.4522, and Egger’s test, p = 0.8167).

**Figure 5:**
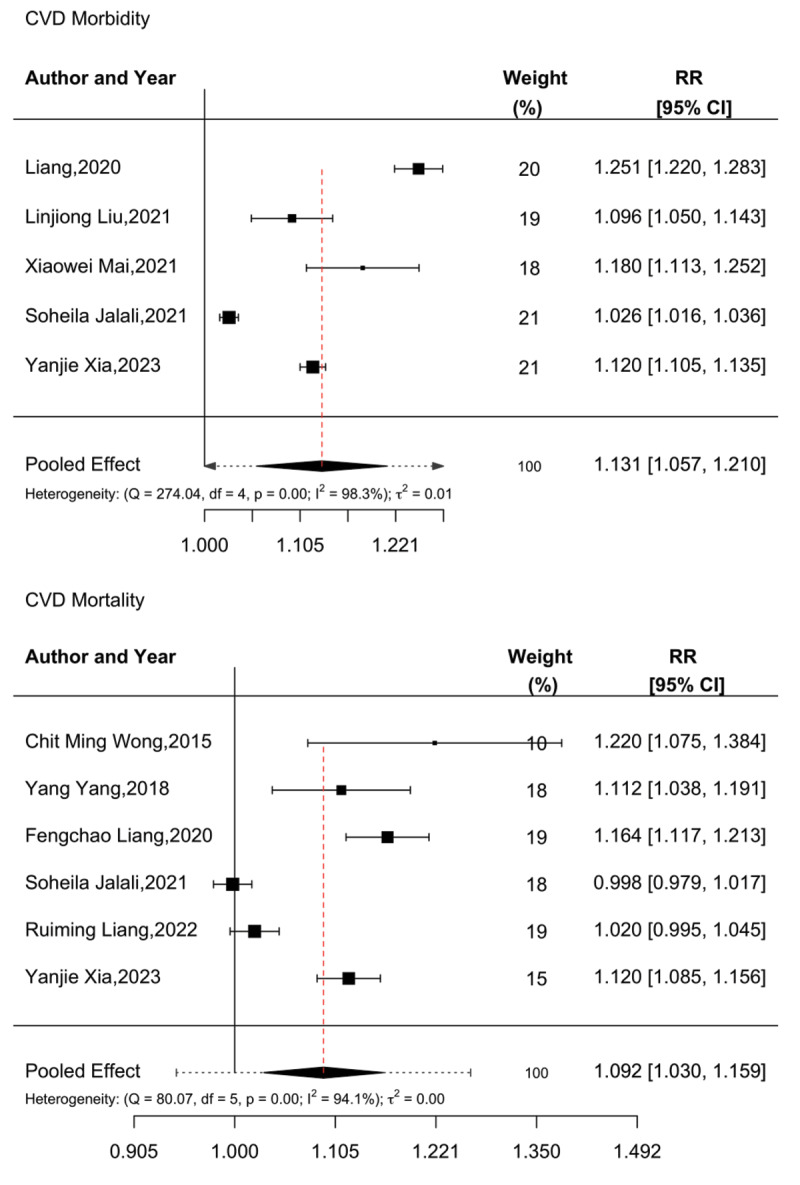
Meta-analysis of long-term PM_2.5_ exposure and cardiovascular disease morbidity and mortality.

All six articles examining mortality also employed cohort design (23,77,79,81,84,85), with meta-analysis indicating that a 10 µg/m^3^ increase in PM_2.5_ exposure increased CVD-related mortality by 9.2% [RR: 1.092 (95% CI: 1.030–1.159)]. However, some evidence of publication bias was observed (Begg’s test, p = 0.0401, and Egger’s test, p = 0.2722). Details are given in Figure 5.

#### 1.2.3: Exposure to SO_2_

One article examined the long-term effects of SO_2_ on CVD morbidity, reporting no association in a cross-sectional analysis (56). In contrast, one article, utilizing a cohort design, investigated the effect of long-term exposure to SO_2_ on CVD mortality, reporting that a 10 µg/m^3^ increase in SO_2_ exposure was associated with a 3.2% (95% CI: 2.3–4.0%) increase in mortality.

#### 1.2.4: Exposure to NO_2_

Two articles assessed the long-term effects of NO_2_ exposure on CVD morbidity (53,56). One, in a cross-sectional analysis, reported no association between NO_2_ and CVD morbidity. By contrast, the second article, employing a cohort design, reported that a 10 µg/m^3^ increase in long-term NO_2_ was associated with a large increase in CVD risk morbidity (RR: 1.558, 95% CI: 1.477–1.642) (53). With respect to mortality, one article examined the impact of NO_2_ exposure on CVD mortality, reporting a relative risk of approximately 1.00.

#### 1.2.5: Exposure to O_3_

Two articles evaluated the long-term effect of O_3_ on CVD morbidity, with a null association observed in cross-sectional analysis (55,56). A cohort study examining O_3_ exposure and CVD morbidity, however, reported that a 10 µg/m^3^ increase in O_3_ was associated with a 7.8% (95% CI: 5.0–10.6) increase in CVD morbidity (55). Two articles assessed the effect of O_3_ on CVD mortality (82,83), both reporting significant associations between O_3_ and CVD mortality, with a 10 µg/m^3^ increase being associated with a 22% (95% CI: 12%–33%) or 9% (95% CI: 4.6%–14.2%) increase in mortality.

#### 1.2.6: Use of solid fuels

Three articles evaluated the long-term effect of using solid/polluting fuels versus clean fuel on CVD morbidity (49,52,54). Two of these studies reported that the long-term use of solid fuels was associated with increased risks of cardiovascular events. One article, examining the implementation of a coal-to-gas/electricity project, did not observe a significant change in cardiovascular morbidity. Two articles examined fuel use and mortality, one examining the use of kerosene in a multi-center study reported that kerosene usage (compared to clean alternatives) was associated with a 34% increase in mortality (95% CI: 8–66%). The other article reported that cooking on solid fuels was associated with a CVD mortality HR of 1.20 (95% CI 1.02–1.41), whereas using them for heating was associated with an HR of 1.29 (95% CI: 1.06–1.55).

#### 1.2.7: Other pollutants

One study assessed the effect of Black Carbon on CVD mortality (50), reporting that an IQR increase in BC (9.6 µg/m^3^) was associated with a 7% (95% CI: 2–11%) increase in CVD mortality.

## Discussion

This review and meta-analysis sought to summarize the available evidence regarding climatic and environmental exposures and their association with CVD morbidity and/or mortality in LMICs. Among the main findings were that both short- and long-term exposure to a variety of air pollutants was associated with both CVD morbidity and mortality. Of note, short- and long-term exposure to PM_2.5_, a well-recognized air contaminant, was consistently associated with increased CVD morbidity and mortality. In addition, short-term disruptions to temperature (both above and below normal) were also associated with morbidity and mortality. Additionally, short-term exposure to NO_2_ and O_3_ was associated with CVD mortality. The long-term use of solid or other polluting fuels was also found to be associated with CVD morbidity and mortality. The vast majority of studies in this review were conducted in China. Other LMIC regions, especially Africa, are significantly understudied, limiting the generalizability of our findings.

Particulate matter (PM) is a commonly used proxy for air pollution. It is a complex mixture of particles that vary widely in size, shape, and chemical composition, which, at smaller sizes, can penetrate the respiratory system. Consistent with other epidemiological studies, we observed PM_2.5_ to be consistently associated with adverse health effects (88). In this review, the overall risk ratio of long-term PM_2.5_ exposure on CVD mortality in LMICs was 1.092 (95% CI: 1.030–1.159) per 10 µg/m^3^ increase. This is comparable to the findings of Guo et al. (2022), who, in their study of LMICs, reported a risk ratio of 1.10 (95% CI: 1.06–1.14) (89) and another study in the United States, which reported a hazard ratio of 1.10 (95% CI: 1.05–1.15) (90).

Many gaseous pollutants such as SO_2_, NO_2_, O_3_, and CO can be classified as short-term exposures due to their brief presence in the atmosphere and temporal variability. Accordingly, the majority of the studies in this review examined the short-term impact of these air pollutants. Of these, NO_2_ and O_3_ were most commonly studied. In general, short-term NO_2_ exposure did not appear to influence CVD morbidity, a finding comparable to findings from high-income countries (91,92,93). In contrast, short-term exposure to NO_2_ was associated with an increase in CVD mortality, with a pooled RR of 1.019 (95% CI: 1.005–1.032), an effect higher than what is seen in higher-income countries (94,95).

A similar phenomenon was observed when examining ozone, where the pooled analysis did not suggest an association with CVD morbidity but did for mortality. This may be indicative of differences in short-term biological effects of these agents or of health care challenges contributing to higher mortality patterns. Compared with results from higher-income countries, this review had divergent results on the effect of ozone on CVD morbidity (92,96).

The use of solid fuels for household heating and cooking is another well-established risk factor for mortality worldwide. The findings from this review largely affirm this finding, showing consistently increased risks of CVD morbidity and mortality in relation to solid fuel use (97,98).

Ongoing climate change means that average temperatures are likely to continue to rise for the foreseeable future. LMICs can be especially vulnerable to the effects of rising temperature, as reflected by the findings from this review, where higher temperatures were consistently associated with increased risks of CVD morbidity and mortality. The overall LMIC research identified in this review showed patterns consistent with findings from high-income countries (99,100,101,102), where both extreme highs and lows of temperature would drive disease morbidity and mortality.

While this review identified nearly 58 articles, a substantial research gap remains, particularly in Sub-Saharan Africa. Most studies identified in this review were conducted in mainland China, which may not accurately reflect other LMICs. The Sub-Saharan Africa region faces distinct climatic/environmental and health challenges, including higher levels of poverty, varying pollution sources, and low-resourced healthcare infrastructures, making increased research crucial. Increased research on environmental health impacts within LMICs, and Sub-Saharan Africa in particular, is crucial for several reasons. First, the sources and types of air pollution can differ by geographic region due to factors such as widespread use of biomass for cooking, dust from unpaved roads, and unregulated emissions from industry. Again, focusing on LMICs can provide comprehensive data for tailored interventions and policies, contributing to more effective public health strategies and global health equity.

The review examined how various environmental exposures affect health in LMICs by examining a wide range of databases. Additionally, this review highlights a major research gap regarding environmental exposure and cardiovascular disease, emphasizing the need for more research, especially in Sub-Saharan African countries. Limitations of this review relate to the relative lack of long-term studies on the effect of climate/air pollution and cardiovascular disease, the lack of studies reporting on specific diagnoses, and the predominance of studies from Asia, hampering our ability to generalize the findings beyond the Asian region. Also, since the study only explored all-cause CVD, we were unable to explore the association of environmental constituents with specific CVD conditions, meaning that specific associations may have been overlooked.

## Conclusion

Short- and long-term exposure to various environmental components was significantly associated with CVD morbidity and mortality in LMICs. Most notably, both short- and long-term exposure to PM_2.5_ was associated with CVD morbidity and mortality, a finding reflected elsewhere in the literature in a variety of settings. In addition, both high and low-temperature extremes were associated with increased morbidity and mortality, and the long-term use of solid (or other polluting) fuels was found to increase CVD mortality. A major research gap was identified where most LMIC research comes from the Asia region, and China in particular, meaning that other regions, especially Sub-Saharan Africa, are markedly understudied. Therefore, context-specific research is needed to understand better the role of environmental disruptions in these understudied regions. Future work will also benefit from examining the association between environmental changes and specific CVD conditions.

## Additional Files

The additional files for this article can be found as follows:

10.5334/gh.1409.s1 Supplementary Document 1. *Review search terms*.

10.5334/gh.1409.s2 Supplementary Figure A1. Meta-analysis of short-term PM_10_ exposure and CVD morbidity and mortality.

10.5334/gh.1409.s3 Supplementary Figure A2. Meta-analysis of SO_2_ exposure and cardiovascular disease morbidity.

## References

[1] Yuyun MF, Sliwa K, Kengne AP, Mocumbi AO, Bukhman G. Cardiovascular diseases in sub-Saharan Africa compared to high-income countries: An epidemiological perspective. Global Heart. 2020;15(1):15. DOI: 10.5334/gh.403 32489788 PMC7218780

[2] Amegah AK. Tackling the growing burden of cardiovascular diseases in sub-Saharan Africa: Need for dietary guidelines. Circulation. 2018;138(22):2449–2451. DOI: 10.1161/CIRCULATIONAHA.118.037367 30571350

[3] World Health Organization (WHO). Cardiovascular diseases (CVDs) [Internet]. World Health Organization; 2021. Available from: https://www.who.int/health-topics/cardiovascular-diseases#tab=tab_1.

[4] UN Environment Programme (UNEP). Pollution Action Note – Data you need to know [Internet]. UN Environment Programme; 2023. Available from: https://www.unep.org/interactives/air-pollution-note/.

[5] World Health Organization (WHO). Air pollution [Internet]. World Health Organization; 2024. Available from: https://www.who.int/health-topics/air-pollution#tab=tab_1.

[6] Shrivastav A, Swetanshu, Singh P. The impact of environmental toxins on cardiovascular diseases. Current Problems in Cardiology. 2023;49:102120. DOI: 10.1016/j.cpcardiol.2023.102120 37805022

[7] United States Environmental Protection Agency (EPA). Air pollution and cardiovascular disease basics [Internet]. United States Environmental Protection Agency; 2024. Available from: https://www.epa.gov/air-research/air-pollution-and-cardiovascular-disease-basics.

[8] Peña MSB, Rollins A. Environmental exposures and cardiovascular disease: A challenge for health and development in low-and middle-income countries. Cardiology Clinics. 2017;35(1):71–86. DOI: 10.1016/j.ccl.2016.09.001 27886791 PMC5129872

[9] Brunekreef B, Holgate ST. Air pollution and health. The Lancet. 2002;360(9341):1233–1242. DOI: 10.1016/S0140-6736(02)11274-8 12401268

[10] Katsouyanni K. Ambient air pollution and health. British Medical Bulletin. 2003;68(1):143–156. DOI: 10.1093/bmb/ldg028 14757714

[11] Landrigan PJ. Air pollution and health. The Lancet Public Health. 2017;2(1):e4–e5. DOI: 10.1016/S2468-2667(16)30023-8 29249479

[12] Schwela D. Air pollution and health in urban areas. Reviews on Environmental Health. 2000;15(1–2):13–42. DOI: 10.1515/REVEH.2000.15.1-2.13 10939084

[13] World Health Organization (WHO). Air quality, energy and health [Internet]. World Health Organization; 2024. Available from: https://www.who.int/teams/environment-climate-change-and-health/air-quality-energy-and-health.

[14] Wong GWK. Air pollution and health. The Lancet Respiratory Medicine. 2014;2(1):8–9. DOI: 10.1016/S2213-2600(13)70284-4 24461884

[15] Nadadur SS, Hollingsworth JW. Air pollution and health effects. London: Springer; 2015. DOI: 10.1007/978-1-4471-6669-6

[16] Lee KK, Bing R, Kiang J, Bashir S, Spath N, Stelzle D, et al. Adverse health effects associated with household air pollution: A systematic review, meta-analysis, and burden estimation study. The Lancet Global Health. 2020;8(11):e1427–e1434. DOI: 10.1016/S2214-109X(20)30343-0 33069303 PMC7564377

[17] Siddharthan T, Grigsby MR, Goodman D, Chowdhury M, Rubinstein A, Irazola V, et al. Association between household air pollution exposure and chronic obstructive pulmonary disease outcomes in 13 low-and middle-income country settings. American Journal of Respiratory and Critical Care Medicine. 2018;197(5):611–620. DOI: 10.1164/rccm.201709-1861OC 29323928 PMC6005243

[18] Khan MN, Nurs CZB, Islam MM, Islam MR, Rahman MM. Household air pollution from cooking and risk of adverse health and birth outcomes in Bangladesh: a nationwide population-based study. Environmental Health. 2017;16:1–8. DOI: 10.1186/s12940-017-0272-y 28610581 PMC5470285

[19] Qui Y, Yang FA, Lai W. The impact of indoor air pollution on health outcomes and cognitive abilities: empirical evidence from China. Population and Environment. 2019;40:388–410. DOI: 10.1007/s11111-019-00317-6

[20] Cheng X, Su H. Effects of climatic temperature stress on cardiovascular diseases. European Journal of Internal Medicine. 2010;21(3):164–167. DOI: 10.1016/j.ejim.2010.03.001 20493415

[21] De Blois J, Kjellstrom T, Agewall S, Ezekowitz JA, Armstrong PW, Atar D. The effects of climate change on cardiac health. Cardiology. 2015;131(4):209–217. DOI: 10.1159/000398787 25997478

[22] Faeh D, Moser A, Panczak R, Bopp M, Röösli M, Spoerri A, et al. Independent at heart: Persistent association of altitude with ischaemic heart disease mortality after consideration of climate, topography and built environment. Journal of Epidemiology and Community Health. 2016;70(8):798–806. DOI: 10.1136/jech-2015-206210 26791518

[23] Jalali S, Karbakhsh M, Momeni M, Taheri M, Amini S, Mansourian M. Long-term exposure to PM 2.5 and cardiovascular disease incidence and mortality in an Eastern Mediterranean country: findings based on a 15-year cohort study. Environmental Health. 2021;20(1):1–16. DOI: 10.1186/s12940-021-00797-w 34711250 PMC8555193

[24] Stewart S, Keates AK, Redfern A, McMurray JJV. Seasonal variations in cardiovascular disease. Nature Reviews Cardiology. 2017;14(11):654–664. DOI: 10.1038/nrcardio.2017.76 28518176

[25] Doku AK, Tetteh J, Edzeame J, Peters RJG, Agyemang C, Otchi EH, et al. The Ghana Heart Initiative–a health system strengthening approach as index intervention model to solving Ghana’s cardiovascular disease burden. Frontiers in Public Health. 2024;12:1330708. DOI: 10.3389/fpubh.2024.1330708 38694980 PMC11061415

[26] Ouzzani M, Hammady H, Fedorowicz Z, Elmagarmid A. Rayyan—a web and mobile app for systematic reviews. Systematic Reviews. 2016;5:1–10. DOI: 10.1186/s13643-016-0384-4 27919275 PMC5139140

[27] van de Schoot R, de Bruin J, Schram R, Zahedi P. An open source machine learning framework for efficient and transparent systematic reviews. Nature Machine Intelligence. 2021;3(2):125–133. DOI: 10.1038/s42256-020-00287-7

[28] Study Quality Assessment Tools. https://www.nhlbi.nih.gov/health-topics/study-quality-assessment-tools. 2017, Accessed.

[29] Begg CB, Mazumdar M. Operating characteristics of a rank correlation test for publication bias. Biometrics. 1994;50(4):1088–1101. DOI: 10.2307/2533446 7786990

[30] R Core Team. R language definition [Internet]. Vienna, Austria: R Foundation for Statistical Computing; 2000. Available from: https://cran.r-project.org/doc/manuals/r-release/R-lang.html.

[31] Giang PN, Dung DV, Giang KB, Vinhc HV, Rocklöv J. The effect of temperature on cardiovascular disease hospital admissions among elderly people in Thai Nguyen Province, Vietnam. Global Health Action. 2014;7(1):23649. DOI: 10.3402/gha.v7.23649 25511886 PMC4265648

[32] Amsalu E, Wang T, Li H, Liu Y, Wang A, Liu X, et al. Acute effects of fine particulate matter (PM 2.5) on hospital admissions for cardiovascular disease in Beijing, China: A time-series study. Environmental Health. 2019;18 1–12. DOI: 10.1186/s12940-019-0506-2 31370900 PMC6670159

[33] Buadong D, Jinsart W, Funatagawa I, Karita K, Yano E. Association between PM_10_ and O_3_ levels and hospital visits for cardiovascular diseases in Bangkok, Thailand. Journal of Epidemiology. 2009;19(4):182–188. DOI: 10.2188/jea.JE20080047 19525614 PMC3924107

[34] Su C, Breitner S, Schneider A, Liu L, Franck U, Peters A, et al. Short-term effects of fine particulate air pollution on cardiovascular hospital emergency room visits: A time-series study in Beijing, China. International Archives of Occupational and Environmental Health. 2016;89(4):641–657. DOI: 10.1007/s00420-015-1102-6 26547916

[35] de Freitas CU, de Leon AP, Juger W, Gouveia N. Air pollution and its impacts on health in Vitoria, Espirito Santo, Brazil. Revista de saude publica. 2016;50:4. DOI: 10.1590/S1518-8787.2016050005909 26982960 PMC4793970

[36] Tong L, Li K, Zhou Q. Promoted relationship of cardiovascular morbidity with air pollutants in a typical Chinese urban area. PLoS One. 2014;9(9):e108076. DOI: 10.1371/journal.pone.0108076 25247693 PMC4172570

[37] Makunyane MS, Rautenbach H, Sweijd N, Botai J, Wichmann J. Health risks of temperature variability on hospital admissions in Cape Town, 2011–2016. International Journal of Environmental Research and Public Health. 2023;20(2):1159. DOI: 10.3390/ijerph20021159 36673914 PMC9859170

[38] Phosri A, Ueda K, Phung VLH, Tawatsupa B, Honda A, Takano H. Effects of ambient air pollution on daily hospital admissions for respiratory and cardiovascular diseases in Bangkok, Thailand. Science of the Total environment. 2019;651:1144–1153. DOI: 10.1016/j.scitotenv.2018.09.183 30360246

[39] Phung D, Hien TT, Linh HN, Luong LMT, Morawska L, Chu C, et al. Air pollution and risk of respiratory and cardiovascular hospitalizations in the most populous city in Vietnam. Science of the Total Environment. 2016;557–558:322–330. DOI: 10.1016/j.scitotenv.2016.03.070 27016680

[40] Rahman MM, Garcia E, Lim CC, Ghazipura M, Alam N, Palinkas LA. Temperature variability associations with cardiovascular and respiratory emergency department visits in Dhaka, Bangladesh. Environment International. 2022;164:107267. DOI: 10.1016/j.envint.2022.107267 35533532 PMC11213361

[41] Zhang J, Chen Q, Wang Q, Ding Z, Sun H, Xu Y. The acute health effects of ozone and PM_2. 5_ on daily cardiovascular disease mortality: A multi-center time series study in China. Ecotoxicology and Environmental Safety. 2019;174:218–223. DOI: 10.1016/j.ecoenv.2019.02.085 30831471

[42] Ma Y, Zhao Y, Yang S, Zhou J, Xin J, Wang S, et al. Short-term effects of ambient air pollution on emergency room admissions due to cardiovascular causes in Beijing, China. Environmental Pollution. 2017;230:974–980. DOI: 10.1016/j.envpol.2017.06.104 28753900

[43] Phosri A, Sihabut T, Jaikanlaya C. Short-term effects of diurnal temperature range on hospital admission in Bangkok, Thailand. Science of the Total environment. 2020;717:137202. DOI: 10.1016/j.scitotenv.2020.137202 32062282

[44] Karbakhsh M, Mansourian M, Taheri M, Rabiei K, Hosseini SM, Rahimi M, et al. Outdoor fine and coarse particles and hospital admissions for cardiovascular diseases: A large-scale case-crossover study. Air Quality, Atmosphere & Health. 2022;15(9):1679–1693. DOI: 10.1007/s11869-022-01212-0

[45] Khan R, Konishi S, Ng CFS, Umezaki M, Kabir AF, Tasmin S, et al. Association between short-term exposure to fine particulate matter and daily emergency room visits at a cardiovascular hospital in Dhaka, Bangladesh. Science of the Total Environment. 2019;646:1030–1036. DOI: 10.1016/j.scitotenv.2018.07.288 30235588

[46] Liu H, Tian Y, Xiang X, Li M, Wu Y, Cao Y, et al. Association of short-term exposure to ambient carbon monoxide with hospital admissions in China. Scientific Reports. 2018;8(1):13336. DOI: 10.1038/s41598-018-31434-1 30190544 PMC6127141

[47] Li H, Wu J, Wang A, Li X, Chen S, Wang T, et al. Effects of ambient carbon monoxide on daily hospitalizations for cardiovascular disease: a time-stratified case-crossover study of 460,938 cases in Beijing, China from 2013 to 2017. Environmental Health. 2018;17:1–11. DOI: 10.1186/s12940-018-0429-3 30477579 PMC6258455

[48] Cheng J, Xu Z, Zhang X, Zhao H, Hu W. Estimating cardiovascular hospitalizations and associated expenses attributable to ambient carbon monoxide in Lanzhou, China: Scientific evidence for policy making. Science of the Total Environment. 2019;682:514–522. DOI: 10.1016/j.scitotenv.2019.05.110 31129539

[49] Ji H, Chen Q, Wu R, Xu J, Chen X, Du L, et al. Indoor solid fuel use for cooking and the risk of incidental non-fatal cardiovascular disease among middle-aged and elderly Chinese adults: a prospective cohort study. BMJ Open. 2022. 12(5):e054170. DOI: 10.1136/bmjopen-2021-054170 PMC9114854 35580969

[50] Liu L, Zhang Y, Yang Z, Luo S, Zhang Y. Long-term exposure to fine particulate constituents and cardiovascular diseases in Chinese adults. Journal of Hazardous Materials. 2021;416:126051. DOI: 10.1016/j.jhazmat.2021.126051 34492892

[51] Mai X, Zhou H, Li Y, Huang X, Yang T. Associations between ambient fine particulate (PM_2. 5_) exposure and cardiovascular disease: findings from the China Health and Retirement Longitudinal Study (CHARLS). Environmental Science and Pollution Research. 2022;29(9):13114–13121. DOI: 10.1007/s11356-021-16541-3 34570321

[52] Wen HX, Nie P, Liu M, Peng R, Guo T, Wang C, et al. Multi-health effects of clean residential heating: evidences from rural China’s coal-to-gas/electricity project. Energy for Sustainable Development. 2023;73:66–75. DOI: 10.1016/j.esd.2023.01.013

[53] Wang K, Yuan Y, Wang Q, Yang Z, Zhan Y, Wang Y. Incident risk and burden of cardiovascular diseases attributable to long-term NO_2_ exposure in Chinese adults. Environment International. 2023;178:108060. DOI: 10.1016/j.envint.2023.108060 37478679

[54] Liu Y, Ning N, Sun T, Guan H, Liu Z, Yang W, et al. Association between solid fuel use and nonfatal cardiovascular disease among middle-aged and older adults: findings from the China health and retirement longitudinal study (CHARLS). Science of the Total Environment. 2023;856:159035. DOI: 10.1016/j.scitotenv.2022.159035 36191716

[55] Zhu L, Fang J, Yao Y, Yang Z, Wu J, Zongwei M, et al. Long-term ambient ozone exposure and incident cardiovascular diseases: National cohort evidence in China. Journal of Hazardous Materials. 2024;471:134158. DOI: 10.1016/j.jhazmat.2024.134158 38636234

[56] Dong GH, Qian ZM, Wang J, Chen W, Ma W, Trevathan E, et al. Associations between ambient air pollution and prevalence of stroke and cardiovascular diseases in 33 Chinese communities. Atmospheric Environment. 2013;77:968–973. DOI: 10.1016/j.atmosenv.2013.06.034

[57] Zhang Y, Huang W, London SJ, Song G, Chen G, Jiang L, et al. Ozone and daily mortality in Shanghai, China. Environmental Health Perspectives. 2006;114(8):1227–1232. DOI: 10.1289/ehp.9014 16882530 PMC1552011

[58] Tam WWS, Wong TW, Chair SY, Wong AHS. Diurnal temperature range and daily cardiovascular mortalities among the elderly in Hong Kong. Archives of Environmental & Occupational Health. 2009;64(3):202–206. DOI: 10.1080/19338240903241192 19864223

[59] Cao J, Yang C, Li J, Chen R, Chen B, Gu D. Association between long-term exposure to outdoor air pollution and mortality in China: A cohort study. Journal of Hazardous Materials. 2011;186(2–3):1594–1600. DOI: 10.1016/j.jhazmat.2010.12.036 21194838

[60] Chen R, Huang W, Wong CM, Wang Z, Thach TQ, Chen B, et al. Short-term exposure to sulfur dioxide and daily mortality in 17 Chinese cities: the China air pollution and health effects study (CAPES). Environmental Research. 2012;118:101–106. DOI: 10.1016/j.envres.2012.07.003 22831556

[61] Geng F, Hua J, Mu Z, Peng L, Xu X, Chen R, et al. Differentiating the associations of black carbon and fine particle with daily mortality in a Chinese city. Environmental Research. 2013;120:27–32. DOI: 10.1016/j.envres.2012.08.007 22981950

[62] Wang C, Chen R, Kuang X, Duan X, Kan H. Temperature and daily mortality in Suzhou, China: a time series analysis. Science of the Total Environment. 2014;466–467:985–990. DOI: 10.1016/j.scitotenv.2013.08.011 23994732

[63] Han J, Liu S, Zhang J, Zhou L, Fang Q, Zhang J, et al. The impact of temperature extremes on mortality: a time-series study in Jinan, China. BMJ Open. 2017;7(4):e014741. DOI: 10.1136/bmjopen-2016-014741 PMC5566622 28465307

[64] Chen C, Zhu P, Lan L, Zhou L, Liu R, Sun Q, et al. Short-term exposures to PM_2.5_ and cause-specific mortality of cardiovascular health in China. Environmental Research. 2018;161:188–194. DOI: 10.1016/j.envres.2017.10.046 29154226

[65] Chen R, Yin P, Meng X, Wang L, Liu C, Niu Y, et al. Associations between ambient nitrogen dioxide and daily cause-specific mortality: evidence from 272 Chinese cities. Epidemiology. 2018;29(4):482–489. DOI: 10.1097/EDE.0000000000000829 29621056

[66] Liu C, Yin P, Chen R, Meng X, Wang L, Niu Y, et al. Ambient carbon monoxide and cardiovascular mortality: a nationwide time-series analysis in 272 cities in China. The Lancet Planetary Health. 2018;2(1):e12–e18. DOI: 10.1016/S2542-5196(17)30181-X 29615203

[67] Wu R, Zhong L, Huang X, Xu H, Liu S, Feng B, et al. Temporal variations in ambient particulate matter reduction associated short-term mortality risks in Guangzhou, China: A time-series analysis (2006–2016). Science of the Total Environment. 2018;645:491–498. DOI: 10.1016/j.scitotenv.2018.07.091 30029124

[68] Liu M, Xue X, Zhou B, Zhang Y, Sun B, Chen J, et al. Population susceptibility differences and effects of air pollution on cardiovascular mortality: Epidemiological evidence from a time-series study. Environmental Science and Pollution Research. 2019;26:15943–15952. DOI: 10.1007/s11356-019-04960-2 30963427

[69] Duan Y, Liao Y, Li H, Yan S, Shao Z, Yu S, et al. Effect of changes in season and temperature on cardiovascular mortality associated with nitrogen dioxide air pollution in Shenzhen, China. Science of the Total Environment. 2019;697:134051. DOI: 10.1016/j.scitotenv.2019.134051 31487586

[70] Iranpour S, Khodakarim S, Shahsavani A, Khosravi A, Etemad K. Modification of the effect of ambient air temperature on cardiovascular and respiratory mortality by air pollution in Ahvaz, Iran. Epidemiology and Health. 2020;42:e2020053. DOI: 10.4178/epih.e2020053 32777886 PMC7871149

[71] Khosravi T, Hadei M, Hopke PK, Namvar Z, Shahsavani A, Nazari SSH, et al. Association of short-term exposure to air pollution with mortality in a middle eastern tourist city. Air Quality, Atmosphere & Health. 2020;13:1223–1234. DOI: 10.1007/s11869-020-00875-x

[72] Zhou H, Geng H, Dong C, Bai T. The short-term harvesting effects of ambient particulate matter on mortality in Taiyuan elderly residents: A time-series analysis with a generalized additive distributed lag model. Ecotoxicology and Environmental Safety. 2021;207:111235. DOI: 10.1016/j.ecoenv.2020.111235 32942099

[73] Li M, Dong H, Wang B, Zhao W, Sakhvidi MJZ, Li L, et al. Association between ambient ozone pollution and mortality from a spectrum of causes in Guangzhou, China. Science of the Total Environment. 2021;754:142110. DOI: 10.1016/j.scitotenv.2020.142110 32920396

[74] Zia Y, Shi C, Li Y, Jiang X, Ruan S, Gao X, et al. Effects of ambient temperature on mortality among elderly residents of Chengdu city in Southwest China, 2016–2020: A distributed-lag non-linear time series analysis. BMC Public Health. 2023;23(1):149. DOI: 10.1186/s12889-022-14931-x 36681785 PMC9863161

[75] Olutola BG, Mwase NS, Shirinde J, Wichmann J. Apparent temperature modifies the effects of air pollution on cardiovascular disease mortality in Cape Town, South Africa. Climate. 2023;11(2):30. DOI: 10.3390/cli11020030

[76] Wichmann J, Voyi K. Ambient air pollution exposure and respiratory, cardiovascular and cerebrovascular mortality in Cape Town, South Africa: 2001–2006. International Journal of Environmental Research and Public Health. 2012;9(11):3978–4016. DOI: 10.3390/ijerph9113978 23202828 PMC3524609

[77] Wong CM, Lai HK, Tsang H, Thach TQ, Thomas GN, Lam KBH, et al. Satellite-based estimates of long-term exposure to fine particles and association with mortality in elderly Hong Kong residents. Environmental Health Perspectives. 2015;123(11):1167–1172. DOI: 10.1289/ehp.1408264 25910279 PMC4629733

[78] Yu K, Qiu G, Chan KH, Lam KBH, Kurmi OP, Bennett DA, et al. Association of solid fuel use with risk of cardiovascular and all-cause mortality in rural China. JAMA. 2018;319(13):1351–1361. DOI: 10.1001/jama.2018.2151 29614179 PMC5933384

[79] Yang Y, Tang R, Qui H, Lai PC, Wong P, Thach TQ, et al. Long term exposure to air pollution and mortality in an elderly cohort in Hong Kong. Environment International. 2018;117:99–106. DOI: 10.1016/j.envint.2018.04.034 29730535

[80] Arku RE, Brauer M, Duong M, Wei L, Hu B, Tse LA, et al. Adverse health impacts of cooking with kerosene: a multi-country analysis within the prospective urban and rural epidemiology study. Environmental Research. 2020;188:109851. DOI: 10.1016/j.envres.2020.109851 32798956 PMC7748391

[81] Liang R, Chen R, Yin P, van Donkelaar A, Martin RV, Burnett R, et al. Associations of long-term exposure to fine particulate matter and its constituents with cardiovascular mortality: A prospective cohort study in China. Environment International. 2022;162:107156. DOI: 10.1016/j.envint.2022.107156 35248978

[82] Liu S, Zhang Y, Ma R, Liu X, Liang J, Lin H, et al. Long-term exposure to ozone and cardiovascular mortality in a large Chinese cohort. Environment International. 2022;165:107280. DOI: 10.1016/j.envint.2022.107280 35605364

[83] Niu Y, Zhou Y, Chen R, Yin P, Meng X, Wang W, et al. Long-term exposure to ozone and cardiovascular mortality in China: a nationwide cohort study. The Lancet Planetary Health. 2022;6(6):e496–e503. DOI: 10.1016/S2542-5196(22)00093-6 35709807

[84] Liang F, Liu F, Huang K, Yang X, Li J, Xiao Q, et al. Long-term exposure to fine particulate matter and cardiovascular disease in China. Journal of the American College of Cardiology. 2020;75(7):707–717. DOI: 10.1016/j.jacc.2019.12.031 32081278

[85] Xia Y, Liu Z, Hu B, Rangarajan S, Tse LA, Li Y, et al. Associations of outdoor fine particulate air pollution and cardiovascular disease: Results from the Prospective Urban and Rural Epidemiology Study in China (PURE-China). Environment International. 2023;174:107829. DOI: 10.1016/j.envint.2023.107829 36934571

[86] Yao C, Wang Y, Williams C, Xu C, Kartsonaki C, Lin Y, et al. The association between high particulate matter pollution and daily cause-specific hospital admissions: a time-series study in Yichang, China. Environmental Science and Pollution Research. 2020;27(5):5240–5250. DOI: 10.1007/s11356-019-06734-2 31848968

[87] Yang C, Yang H, Guo S, Wang Z, Xu X, Duan X, et al. Alternative ozone metrics and daily mortality in Suzhou: the China Air Pollution and Health Effects Study (CAPES). Science of the Total Environment. 2012;426:83–89. DOI: 10.1016/j.scitotenv.2012.03.036 22521098

[88] Lee BJ, Kim B, Lee K. Air pollution exposure and cardiovascular disease. Toxicological Research. 2014;30(2):71–75. DOI: 10.5487/TR.2014.30.2.071 25071915 PMC4112067

[89] Guo J, Chai G, Song X, Hui X, Li Z, Feng X, et al. Long-term exposure to particulate matter on cardiovascular and respiratory diseases in low-and middle-income countries: A systematic review and meta-analysis. Frontiers in Public Health. 2023;11:1134341. DOI: 10.3389/fpubh.2023.1134341 37056647 PMC10089304

[90] Sharma S, Chandra M, Kota SH. Health effects associated with PM 2.5: A systematic review. Current Pollution Reports. 2020;6:345–367. DOI: 10.1007/s40726-020-00155-3

[91] Mills IC, Atkinson RW, Kang S, Walton H, Anderson HR. Quantitative systematic review of the associations between short-term exposure to nitrogen dioxide and mortality and hospital admissions. BMJ Open. 2015;5(5):e006946. DOI: 10.1136/bmjopen-2014-006946 PMC4452753 25967992

[92] Chen Z, Liu N, Tang H, Gao X, Zhang Y, Kan H, et al. Health effects of exposure to sulfur dioxide, nitrogen dioxide, ozone, and carbon monoxide between 1980 and 2019: A systematic review and meta-analysis. Indoor Air. 2022;32(11):e13170. DOI: 10.1111/ina.13170 36437665

[93] Collart P, Dubourg D, Levêque A, Sierra NB, Coppieters Y. Short-term effects of nitrogen dioxide on hospital admissions for cardiovascular disease in Wallonia, Belgium. International Journal of Cardiology. 2018;255:231–236. DOI: 10.1016/j.ijcard.2017.12.058 29288056

[94] Wang M, Li H, Huang S, Qian Y, Steenland K, Xie Y, et al. Short-term exposure to nitrogen dioxide and mortality: a systematic review and meta-analysis. Environmental Research. 2021;202:111766. DOI: 10.1016/j.envres.2021.111766 34331919 PMC8578359

[95] Anderson HR, Atkinson RW, Bremner SA, Carrington J, Peacock J. Quantitative systematic review of short term associations between ambient air pollution (particulate matter, ozone, nitrogen dioxide, sulphur dioxide and carbon monoxide), and mortality and morbidity. London: Division of Community Health Sciences, St. George’s University of London; 2007 June. 706 p.

[96] Wu K, Ho HC, Su H, Huang C, Zheng H, Zhang W, et al. A systematic review and meta-analysis of intraday effects of ambient air pollution and temperature on cardiorespiratory morbidities: First few hours of exposure matters to life. EBio Medicine. 2022;86:104327. DOI: 10.1016/j.ebiom.2022.104327 PMC9626385 36323182

[97] Huang S, Li H, Wang M, Qian Y, Steenland K, Caudle WM, et al. Long-term exposure to nitrogen dioxide and mortality: A systematic review and meta-analysis. Science of the Total Environment. 2021;776:145968. DOI: 10.1016/j.scitotenv.2021.145968 33640547 PMC8499020

[98] Hystad P, Duong M, Brauer M, Larkin A, Arku R, Kurmi OP, et al. Health effects of household solid fuel use: findings from 11 countries within the prospective urban and rural epidemiology study. Environmental Health Perspectives. 2019;127(5):057003. DOI: 10.1289/EHP3915 31067132 PMC6791569

[99] Moghadamnia MT, Ardalan A, Mesdaghinia A, Keshtkar A, Naddafi K, Yekaninejad MS. Ambient temperature and cardiovascular mortality: a systematic review and meta-analysis. PeerJ. 2017;5:e3574. DOI: 10.7717/peerj.3574 28791197 PMC5546177

[100] Phung D, Thai PK, Guo Y, Morawska L, Rutherford S, Chu C. Ambient temperature and risk of cardiovascular hospitalization: An updated systematic review and meta-analysis. Science of the Total Environment. 2016;550:1084–1102. DOI: 10.1016/j.scitotenv.2016.01.154 26871555

[101] Moslehi S, Dowlati M. Effects of extreme ambient temperature on cardiovascular outcomes: A systematic review. Journal of Environmental Health and Sustainable Development. 2021;6(4):1407–1418. DOI: 10.18502/jehsd.v6i4.8148

[102] Fan JF, Xiao YC, Feng YF, Niu LY, Tan X, Sun JC, et al. A systematic review and meta-analysis of cold exposure and cardiovascular disease outcomes. Frontiers in Cardiovascular Medicine. 2023;10:1084611. DOI: 10.3389/fcvm.2023.1084611 37051068 PMC10083291

