# Supplementary material for: Climate, Air Quality and Their Contribution to Cardiovascular Disease Morbidity and Mortality in Low- and Middle-Income Countries: A Systematic Review and Meta-Analysis
Source: Glob Heart. 2025 Mar 27;20(1):35. doi: 10.5334/gh.1409 (PMC11951997; doi:10.5334/gh.1409)
Supplement: Supplementary Document 1. — Review search terms. [file gh-20-1-1409-s1.pdf]

Supplementary Document 1: *Review search terms*

PubMed

(“cardiovascular disease*”[title/abstract] OR “cardiovascular diseases”[Mesh] OR cardiovascular morbidit*[title/abstract] OR “cardiovascular illness*”[title/abstract] OR "Cardiovascular Abnormalit*"[title/abstract] OR "Cardiovascular Abnormalities"[Mesh] OR "Heart Disease*"[title/abstract] OR "Heart Diseases"[Mesh] OR "Noncommunicable Diseases"[title/abstract] OR "Noncommunicable Diseases"[Mesh] OR “cardiac disease*”[title/abstract] OR "Cardiometabolic disease*"[title/abstract] OR "Coronary Disease*"[title/abstract] OR "Coronary Disease"[Mesh] OR "Cerebrovascular Disorder*"[title/abstract] OR "Cerebrovascular Disorders"[Mesh] OR "Peripheral Arterial Disease*"[title/abstract] OR "Peripheral Arterial Disease"[Mesh] OR "Rheumatic Heart Disease*"[title/abstract] OR "Rheumatic Heart Disease"[Mesh] OR "Venous Thrombosis"[title/abstract] OR "Venous Thrombosis"[Mesh] OR "Pulmonary Embolism"[title/abstract] OR "Pulmonary Embolism"[Mesh] OR “cardiovascular hospitalizat*”[title/abstract] OR "heart disease morbidity"[title/abstract] OR "Coronary disease morbidity"[title/abstract] OR "Cerebrovascular morbidit*"[title/abstract] OR "Angina"[title/abstract] OR "Angina Pectoris"[Mesh] OR "Myocardial Infarction"[title/abstract] OR "Myocardial Infarction"[Mesh] OR “Ischemic heart disease”[title/abstract] OR “Ischemic heart disease morbidity”[title/abstract] OR "Myocardial Ischemia"[Mesh] OR “Aneurysm"[title/abstract] OR "Aneurysm"[Mesh] OR "Hypertension"[title/abstract] OR "Hypertension"[Mesh] OR “heart attack”[title/abstract] OR “heart failure”[title/abstract] OR "Heart Failure"[Mesh] OR “high blood pressure”[title/abstract]) OR ("cardiovascular mortalit*"[Title/Abstract] OR "cardiovascular death"[Title/Abstract] OR "cardiovascular disease death"[title/abstract] OR "cardiovascular disease mortalit*"[title/abstract] OR "death, sudden, cardiac"[title/abstract] OR "death, sudden, cardiac"[MeSH] OR "heart disease mortality"[title/abstract] OR "Coronary disease mortality"[title/abstract] OR "Cerebrovascular mortality"[title/abstract] OR "Rheumatic Heart disease mortality"[title/abstract] OR "Venous Thrombosis mortality"[title/abstract] OR "Pulmonary Embolism mortality"[title/abstract] OR "mortality" [Subheading] OR “Ischemic heart disease mortality”[title/abstract] OR “Aneurysm mortality”[title/abstract] OR “hypertension mortality”[title/abstract] OR “heart failure”[title/abstract]) AND ("climat*"[Title/Abstract] OR "Climate"[MeSH] OR "Climate Change"[title/abstract] OR "Climate Change"[MeSH] OR "humid*"[Title/Abstract] OR "Humidity"[MeSH] OR "temperature*"[Title/Abstract] OR "Temperature"[MeSH] OR "Weather"[title/abstract] OR "Weather"[MeSH] OR "Extreme Cold Weather"[title/abstract] OR "Extreme Cold Weather"[MeSH] OR "Extreme Weather"[title/abstract] OR "Extreme Weather"[MeSH] OR "Wind"[title/abstract] OR "Wind"[MeSH] OR "Air"[title/abstract] OR "Air"[MeSH] OR "Climatic Processes"[title/abstract] OR "Climatic Processes"[MeSH] OR "Environment"[title/abstract] OR "Environment"[MeSH] OR "Solar Energy"[title/abstract] OR "Solar Energy"[MeSH] OR "PM2.5"[Title/Abstract] OR "household pollut*"[Title/Abstract] OR "household air pollut*"[Title/Abstract] OR "HAP"[Title/Abstract] OR "pollut*"[Title/Abstract] OR "household energy"[Title/Abstract] OR "cookstove*"[Title/Abstract] OR "Air Pollut*"[title/abstract] OR "Air Pollution"[MeSH] OR "Particulate Matter"[title/abstract] OR "Particulate Matter"[MeSH] OR "Tobacco Smoke Pollution"[title/abstract] OR "Tobacco Smoke Pollution"[MeSH] OR "Sanitation"[title/abstract] OR "Sanitation"[MeSH] OR "Environmental Exposure"[title/abstract] OR "Environmental Exposure"[MeSH])

Filters

("developing countr*"[title/abstract] OR “middle income countr*”[title/abstract] OR "developing countries"[MeSH] OR “developing population”[title/abstract] OR “developing

econom*”[title/abstract] OR “middle income nation”[title/abstract] OR “middle income population”[title/abstract] OR “middle income econom*”[title/abstract] OR “middle resource setting”[title/abstract] OR ("developing"[All Fields] AND "countries"[All Fields]) OR "developing countries"[All Fields] OR ("middle"[All Fields] AND "income"[All Fields] AND "countries"[All Fields]) OR "middle income countries"[All Fields] OR “LMIC”[title/abstract] OR “low middle income countr*”[title/abstract] OR “low income population”[title/abstract] OR “low resource setting”[title/abstract] OR “low income nation*”[title/abstract] OR “low income econom*”[title/abstract]) AND **("Adult"[tiab] OR "young adult"[tiab] OR “middle aged”[tiab] OR "very elderly"[tiab] OR "above 18 years"[tiab] OR "adult"[MeSH])**

EMBASE

‘cardiovascular disease*’:ti,ab,kw OR ‘cardiovascular diseases’/exp OR cardiovascular morbidit*:ti,ab,kw OR ‘cardiovascular illness*’:ti,ab,kw OR ‘Cardiovascular Abnormalit*’:ti,ab,kw OR ‘Cardiovascular Abnormalities’/exp OR ‘Heart Disease*’:ti,ab,kw OR ‘Heart Diseases’/exp OR ‘Noncommunicable Diseases’:ti,ab,kw OR ‘Noncommunicable Diseases’/exp OR ‘cardiac disease*’:ti,ab,kw OR ‘Cardiometabolic disease*’:ti,ab,kw OR ‘Coronary Disease*’:ti,ab,kw OR ‘Coronary Disease’/exp OR ‘Cerebrovascular Disorder*’:ti,ab,kw OR ‘Cerebrovascular Disorders’/exp OR ‘Peripheral Arterial Disease*’:ti,ab,kw OR ‘Peripheral Arterial Disease’/exp OR ‘Rheumatic Heart Disease*’:ti,ab,kw OR ‘Rheumatic Heart Disease’/exp OR ‘Venous Thrombosis’:ti,ab,kw OR ‘Venous Thrombosis’/exp OR ‘Pulmonary Embolism’:ti,ab,kw OR ‘Pulmonary Embolism’/exp OR ‘cardiovascular hospitalizat*’:ti,ab,kw OR ‘heart disease morbidity’:ti,ab,kw OR ‘Coronary disease morbidity’:ti,ab,kw OR ‘Cerebrovascular morbidit*’:ti,ab,kw OR ‘Angina’:ti,ab,kw OR ‘Angina Pectoris’/exp OR ‘Myocardial Infarction’:ti,ab,kw OR ‘Myocardial Infarction’/exp OR ‘Ischemic heart disease’:ti,ab,kw OR ‘Ischemic heart disease morbidity’:ti,ab,kw OR ‘Myocardial Ischemia’/exp OR ‘Aneurysm’:ti,ab,kw OR ‘Aneurysm’/exp OR ‘hypertension’:ti,ab,kw OR ‘hypertension’/exp OR ‘heart failure’:ti,ab,kw OR ‘heart failure’/exp OR ‘heart attacks’:ti,ab,kw OR ‘high blood pressure’:ti,ab,kw) OR ‘cardiovascular mortalit*’:ti,ab,kw OR ‘cardiovascular death’:ti,ab,kw OR ‘cardiovascular disease death’:ti,ab,kw OR ‘cardiovascular disease mortalit*’:ti,ab,kw OR ‘cardiovascular mortality’/exp OR ‘death, sudden, cardiac’:ti,ab,kw OR ‘death, sudden, cardiac’/exp OR ‘heart disease mortality’:ti,ab,kw OR ‘Coronary disease mortality’:ti,ab,kw OR ‘Cerebrovascular mortality’:ti,ab,kw OR ‘Rheumatic Heart disease mortality’:ti,ab,kw OR ‘Venous Thrombosis mortality’:ti,ab,kw OR ‘Pulmonary Embolism mortality’:ti,ab,kw OR ‘Ischemic heart disease mortality’:ti,ab,kw OR ‘Aneurysm mortality’:ti,ab,kw OR ‘hypertension mortality’:ti,ab,kw ‘climat*’:ti,ab,kw OR ‘Climate’/exp OR ‘Climate Change’:ti,ab,kw OR ‘Climate Change’/exp OR ‘humid*’:ti,ab,kw OR ‘humidity’/exp OR ‘temperature*’:ti,ab,kw OR ‘Temperature’/exp OR ‘Weather’:ti,ab,kw OR ‘Weather’/exp OR ‘Extreme Cold Weather’:ti,ab,kw OR ‘extreme cold weather’/exp OR ‘extreme weather’:ti,ab,kw OR ‘extreme weather’/exp OR ‘Wind’:ti,ab,kw OR ‘Wind’/exp OR ‘Air’:ti,ab,kw OR ‘Air’/exp OR ‘Environment’:ti,ab,kw OR ‘environment’/exp OR ‘Solar Energy’:ti,ab,kw OR ‘Solar Energy’/exp OR ‘PM2.5’:ti,ab,kw OR ‘household pollut*’:ti,ab,kw OR ‘household air pollution’:ti,ab,kw OR ‘pollut*’:ti,ab,kw OR ‘household energy’:ti,ab,kw OR ‘cookstove*’:ti,ab,kw OR ‘Air Pollut*’:ti,ab,kw OR ‘Air Pollution’/exp OR ‘Particulate Matter’:ti,ab,kw OR ‘Particulate Matter’/exp OR ‘Tobacco Smoke Pollution’:ti,ab,kw OR ‘Tobacco Smoke Pollution’/exp OR ‘Sanitation’:ti,ab,kw OR ‘Sanitation’/exp OR ‘Environmental Exposure’:ti,ab,kw OR ‘Environmental Exposure’/exp

Filters

‘developing countr*’:ti,ab,kw OR ‘middle income countr*’:ti,ab,kw OR ‘developing countries’/exp OR ‘developing population’:ti,ab,kw OR ‘developing econom*’:ti,ab,kw OR

‘middle income nation’:ti,ab,kw OR ‘middle income population’:ti,ab,kw OR ‘middle income econom*’:ti,ab,kw OR ‘middle resource setting’:ti,ab,kw OR ‘LMIC’:ti,ab,kw OR ‘low middle income countr*’:ti,ab,kw OR ‘low income population’:ti,ab,kw OR ‘low resource setting’:ti,ab,kw OR ‘low income nation*’:ti,ab,kw OR ‘low income econom*’:ti,ab,kw) AND 'adult*':ti,ab,kw OR 'young adult':ti,ab,kw OR 'very elderly':ti,ab,kw OR ‘middle aged’:ti,ab,kw OR 'above 18 years':ti,ab,kw OR 'adult'/exp OR ‘very elderly’/exp

SCOPUS

TITLE-ABS-KEY(((“cardiovascular disease*” OR cardiovascular morbidit* OR “cardiovascular illness*” OR "Cardiovascular Abnormalit*" OR "Heart Disease*" OR "Noncommunicable Diseases" OR “cardiac disease*” OR "Cardiometabolic disease*" OR "Coronary Disease*" OR "Cerebrovascular Disorder*" OR "Peripheral Arterial Disease*" OR "Rheumatic Heart Disease*" OR "Venous Thrombosis" OR "Pulmonary Embolism" OR “cardiovascular hospitalizat*” OR "heart disease morbidity" OR "Coronary disease morbidity" OR "Cerebrovascular morbidit*" OR "Angina" OR "Myocardial Infarction" OR “Ischemic heart disease” OR “Ischemic heart disease morbidity” OR “Aneurysm" OR "Hypertension" OR “heart attack” OR “heart failure” OR “high blood pressure”)) OR TITLE-ABS-KEY ("cardiovascular mortalit*" OR "cardiovascular death" OR "cardiovascular disease death" OR "cardiovascular disease mortalit*" OR "death, sudden, cardiac" OR "heart disease mortality" OR "Coronary disease mortality" OR "Cerebrovascular mortality" OR "Rheumatic Heart disease mortality" OR "Venous Thrombosis mortality" OR "Pulmonary Embolism mortality" OR "mortality" [Subheading] OR “Ischemic heart disease mortality” OR “Aneurysm mortality” OR “hypertension mortality” OR “heart failure”)) AND TITLE-ABS-KEY ("climat*" OR "Climate Change" OR "humid*" OR "temperature*" OR "Weather" OR "Extreme Cold Weather" OR "Extreme Weather" OR "Wind" OR "Air" OR "Climatic Processes" OR "Environment" OR "Solar Energy" OR "PM2.5" OR "household pollut*" OR "household air pollut*" OR "HAP" OR "pollut*" OR "household energy" OR "cookstove*" OR "Air Pollut*" OR "Particulate Matter" OR "Tobacco Smoke Pollution" OR "Sanitation" OR "Environmental Exposure")

Filters

TITLE-ABS-KEY (("developing countr*" OR "middle income countr*" OR "developing population” OR "developing econom*" OR “developing nation*” OR “middle income nation*” OR “middle income population” OR “middle income econom*” OR “middle resource setting” OR “LMIC” OR “low income population” OR “low resource setting” OR “low income nation*” OR “low income econom*”) OR (“developing countries”[all AND fields] OR “middle income countries”[all AND fields]))AND TITLE-ABS-KEY (("adult” OR “young adult” OR “very elderly” OR “middle aged” OR “above 18 years”) OR (“adult”[all AND fields] OR “young adult”[all and fields]))

Web of Science

ALL=(“cardiovascular disease” OR “cardiovascular diseases” OR “cardiovascular morbidity” OR “cardiovascular morbidities” OR “cardiovascular illness” OR “heart disease” OR “noncommuicable disease” OR “cardiac disease” OR "Cardiometabolic disease" OR “Coronary Disease” OR “Coronary Diseases” OR “Cerebrovascular Disorder” OR “Peripheral Arterial Disease” OR “Rheumatic Heart Disease” OR “Venous Thrombosis” OR “Pulmonary Embolism” OR “cardiovascular hospitalize” OR “cardiovascular hospitalization” OR “heart disease morbidity” OR “heart disease morbidities” OR “Coronary disease morbidity” OR “Coronary disease morbidities” OR “Cerebrovascular morbidity” OR

“Cerebrovascular morbidities” OR “Angina Pectoris” OR “Myocardial Infarction” OR “Ischemic heart disease” OR “Ischemic heart disease morbidity” OR “Myocardial Ischemia” OR “Aneurysm” OR hypertension OR “heart attack” OR “heart failure” OR “high blood pressure”) OR ALL=(“cardiovascular mortality” OR “cardiovascular mortalities” OR “cardiovascular death” OR “cardiovascular disease death” OR “cardiovascular disease mortality” OR “death, sudden, cardiac” OR “heart disease mortality” OR “Coronary disease mortality” OR “Cerebrovascular mortality” OR “Rheumatic Heart disease mortality” OR “Venous Thrombosis mortality” OR “Pulmonary Embolism mortality” OR “Ischemic heart disease mortality” OR “Aneurysm mortality” OR “hypertension death” OR “hypertension mortality” OR “heart failure death” OR “heart failure mortality” OR “heat failure death” OR “heart failure mortality”) AND ALL= (“climate” OR “Climate Change” OR “humidity” OR “humid” OR “temperature” OR “Weather” OR “Extreme Cold Weather” OR “extreme weather” OR “Wind” OR “Air” OR “Environment” OR “Solar Energy” OR “PM2.5” OR “household pollution” OR “household air pollution” OR “pollution” OR “household energy” OR “cookstove” OR “Air Pollution” OR “Particulate Matter” OR “Tobacco Smoke Pollution” OR “Sanitation” OR “Environmental Exposure” OR “Environment”) AND ALL= ("developing country" OR “developing countries” OR “middle income country” OR “middle income countries” OR “developing population” OR “developing economy” OR “developing economies” OR “middle income nation” OR “middle income population” OR “middle income economy” OR “middle income economies” OR “middle resource setting” OR “LMIC” OR “low middle income country” OR “low middle income countries” OR “low income population” OR “low resource setting” OR “low income nation” OR “low income economy” OR “low income economies”) AND ALL= ("adult” OR “young adult” OR “very elderly” OR “middle aged” OR “above 18 years”)

GLOBAL HEALTH

(((“cardiovascular diseas*” OR “cardiovascular morbidit*” OR “cardiovascular illness” OR “heart disease” OR “noncommuicable disease” OR “cardiac disease” OR "Cardiometabolic disease" OR “Coronary Disease” OR “Coronary Diseases” OR “Cerebrovascular Disorder” OR “Peripheral Arterial Disease” OR “Rheumatic Heart Disease” OR “Venous Thrombosis” OR “Pulmonary Embolism” OR “cardiovascular hospitaliz*” OR “heart disease morbidit*” OR “Coronary disease morbidit*” OR “Cerebrovascular morbidit*” OR “Angina Pectoris” OR “Myocardial Infarction” OR “Ischemic heart disease” OR “Ischemic heart disease morbidity” OR “Myocardial Ischemia” OR “Aneurysm” OR hypertension OR “heart attack” OR “heart failure” OR “high blood pressure”) OR (“cardiovascular mortalit*” OR “cardiovascular death” OR “cardiovascular disease death” OR “cardiovascular disease mortality” OR “death, sudden, cardiac” OR “heart disease mortality” OR “Coronary disease mortality” OR “Cerebrovascular mortality” OR “Rheumatic Heart disease mortality” OR “Venous Thrombosis mortality” OR “Pulmonary Embolism mortality” OR “Ischemic heart disease mortality” OR “Aneurysm mortality” OR “hypertension death” OR “hypertension mortality” OR “heart failure death” OR “heart failure mortality” OR “heat failure death” OR “heart failure mortality”)) AND (“climate” OR “Climate Change” OR “humid*” OR “temperature” OR “Weather” OR “Extreme Cold Weather” OR “extreme weather” OR “Wind” OR “Air” OR “Environment” OR “Solar Energy” OR “PM2.5” OR “household pollut*” OR “household air pollut*” OR “pollut*” OR “household energy” OR “cookstove” OR “Air Pollution” OR “Particulate Matter” OR “Tobacco Smoke Pollution” OR “Sanitation” OR “Environmental Exposure” OR “Environment”) AND ("developing countr*" OR “middle income countr*” OR “developing population” OR “developing econom*” OR “middle income nation” OR “middle income population” OR “middle income econom*” OR “middle resource setting” OR “LMIC” OR “low middle income countr*” OR “low income population” OR “low resource setting” OR “low income nation” OR “low income econom*”)

AND ("adult" OR "young adult" OR "very elderly" OR "middle aged" OR "above 18 years")

LILACS

tw:(((cardiovascular disease) OR (cardiovascular morbidity) OR (cardiovascular illness) OR (heart disease) OR (noncommuicable disease) OR (NCD) OR (cardiac disease) OR (Cardiometabolic disease) OR (Coronary Disease) OR (Cerebrovascular Disorder) OR (Peripheral Arterial Disease) OR (Rheumatic Heart Disease) OR (Venous Thrombosis) OR (Pulmonary Embolism) OR (cardiovascular hospitalization) OR (heart disease morbidity) OR (Coronary disease morbidity) OR (Cerebrovascular morbidity) OR (Angina Pectoris) OR (Myocardial Infarction) OR (Ischemic heart disease) OR (Ischemic heart disease morbidity) OR (Myocardial Ischemia) OR (Aneurysm) OR (hypertension) OR (heart attack) OR (heart failure) OR (high blood pressure)) OR ((cardiovascular mortality) OR (cardiovascular death) OR (cardiovascular disease death) OR (cardiovascular disease mortality) OR (death, sudden, cardiac) OR (heart disease mortality) OR (Coronary disease mortality) OR (Cerebrovascular mortality) OR (Rheumatic Heart disease mortality) OR (Venous Thrombosis mortality) OR (Pulmonary Embolism mortality) OR (Ischemic heart disease mortality) OR (Aneurysm mortality) OR (hypertension death) OR (hypertension mortality) OR (heart failure death) OR (heart failure mortality) OR (heat failure death) OR (heart failure mortality)) AND ((climate) OR (climate change) OR (humidity) OR (temperature) OR (PM2.5) OR (household pollution) OR (household air pollution) OR (HAP) OR (pollution) OR (household energy) OR (cookstove) OR (Weather) OR (Extreme Cold Weather) OR (Wind) OR (Air) OR (Climatic Processes) OR (Environment) OR (Solar Energy) OR (Particulate Matter) OR (Tobacco Smoke Pollution) OR (Working Conditions) OR (Neighborhood Characteristics) OR (Sanitation) OR (Environmental Exposure))) AND tw:((developing country) OR (middle income country) OR (developing population) OR (developing economy) OR (middle income nation) OR (middle income population) OR (middle income economy) OR (middle resource setting) OR (LMIC) OR (low middle income country) OR (low income population) OR (low resource setting) OR (low income nation) OR (low income economy)) AND tw:((adult) OR (young adult) OR (very elderly) OR (middle aged) OR (above 18 years))

AFICAN INDEX MEDICUS (AIM)

tw:(((cardiovascular disease) OR (cardiovascular morbidity) OR (cardiovascular illness) OR (heart disease) OR (noncommuicable disease) OR (NCD) OR (cardiac disease) OR (Cardiometabolic disease) OR (Coronary Disease) OR (Cerebrovascular Disorder) OR (Peripheral Arterial Disease) OR (Rheumatic Heart Disease) OR (Venous Thrombosis) OR (Pulmonary Embolism) OR (cardiovascular hospitalization) OR (heart disease morbidity) OR (Coronary disease morbidity) OR (Cerebrovascular morbidity) OR (Angina Pectoris) OR (Myocardial Infarction) OR (Ischemic heart disease) OR (Ischemic heart disease morbidity) OR (Myocardial Ischemia) OR (Aneurysm) OR (hypertension) OR (heart attack) OR (heart failure) OR (high blood pressure)) OR ((cardiovascular mortality) OR (cardiovascular death) OR (cardiovascular disease death) OR (cardiovascular disease mortality) OR (death, sudden, cardiac) OR (heart disease mortality) OR (Coronary disease mortality) OR (Cerebrovascular mortality) OR (Rheumatic Heart disease mortality) OR (Venous Thrombosis mortality) OR (Pulmonary Embolism mortality) OR (Ischemic heart disease mortality) OR (Aneurysm mortality) OR (hypertension death) OR (hypertension mortality) OR (heart failure death) OR (heart failure mortality) OR (heat failure death) OR (heart failure mortality)) AND ((climate) OR (climate change) OR (humidity) OR (temperature) OR (PM2.5) OR (household pollution) OR (household air pollution) OR (HAP) OR (pollution) OR (household energy) OR (cookstove) OR (Weather) OR (Extreme Cold Weather) OR (Wind) OR (Air) OR (Climatic Processes) OR (Environment) OR (Solar Energy) OR (Particulate Matter) OR (Tobacco Smoke Pollution) OR (Working Conditions) OR (Neighborhood Characteristics) OR

(Sanitation) OR (Environmental Exposure))) AND tw:((adult) OR (young adult) OR (very elderly) OR (middle aged) OR (above 18 years))
