# Supplementary figures and images for: Climate, Air Quality and Their Contribution to Cardiovascular Disease Morbidity and Mortality in Low- and Middle-Income Countries: A Systematic Review and Meta-Analysis
Source: Glob Heart. 2025 Mar 27;20(1):35. doi: 10.5334/gh.1409 (PMC11951997; doi:10.5334/gh.1409)

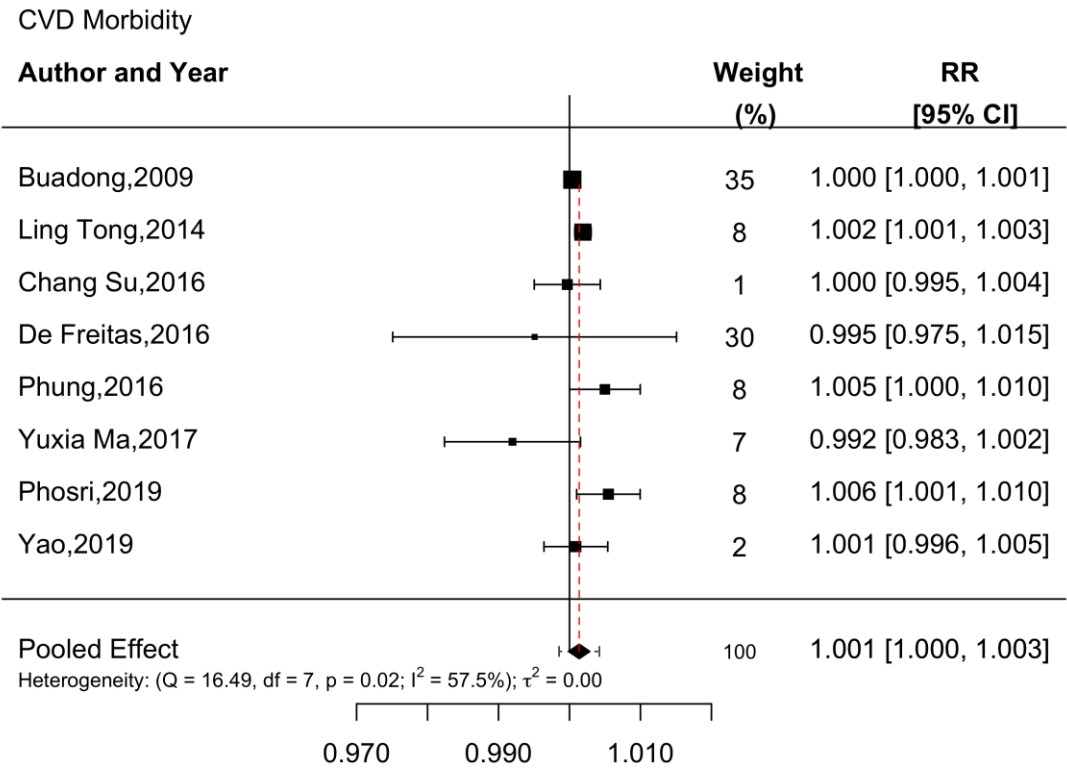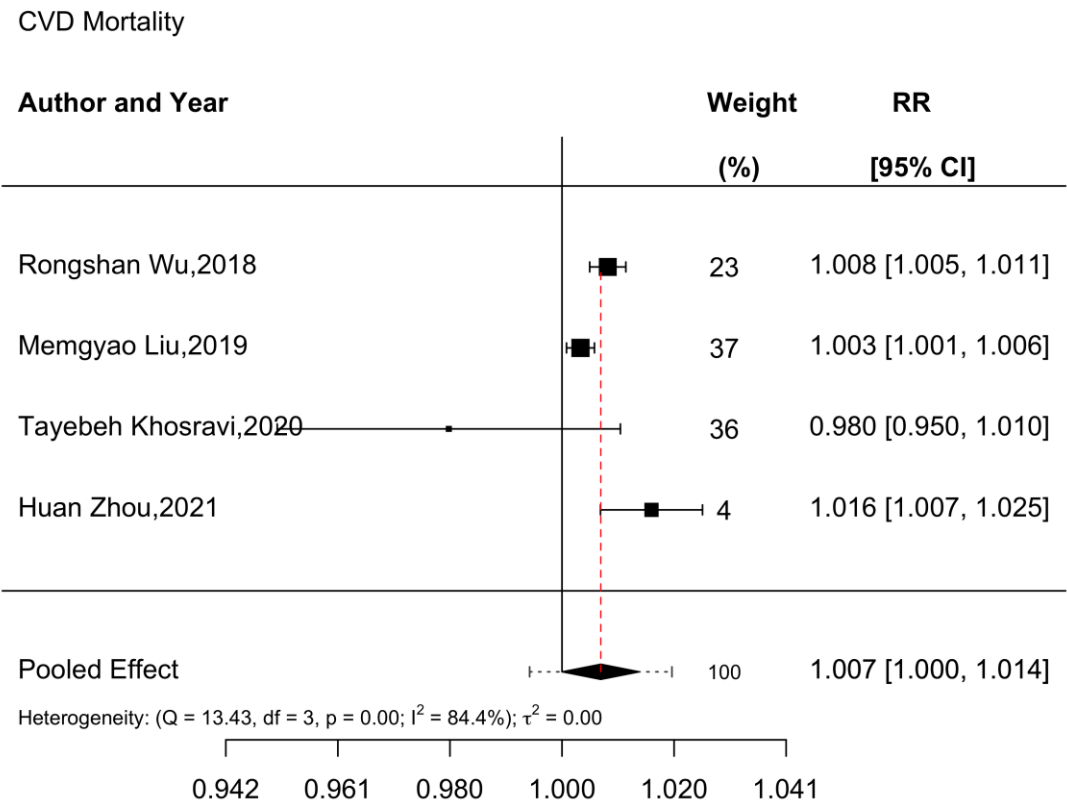

Supplement: Supplementary Figure A1. — Meta-analysis of short-term PM10 exposure and CVD morbidity and mortality. [file gh-20-1-1409-s2.pdf]

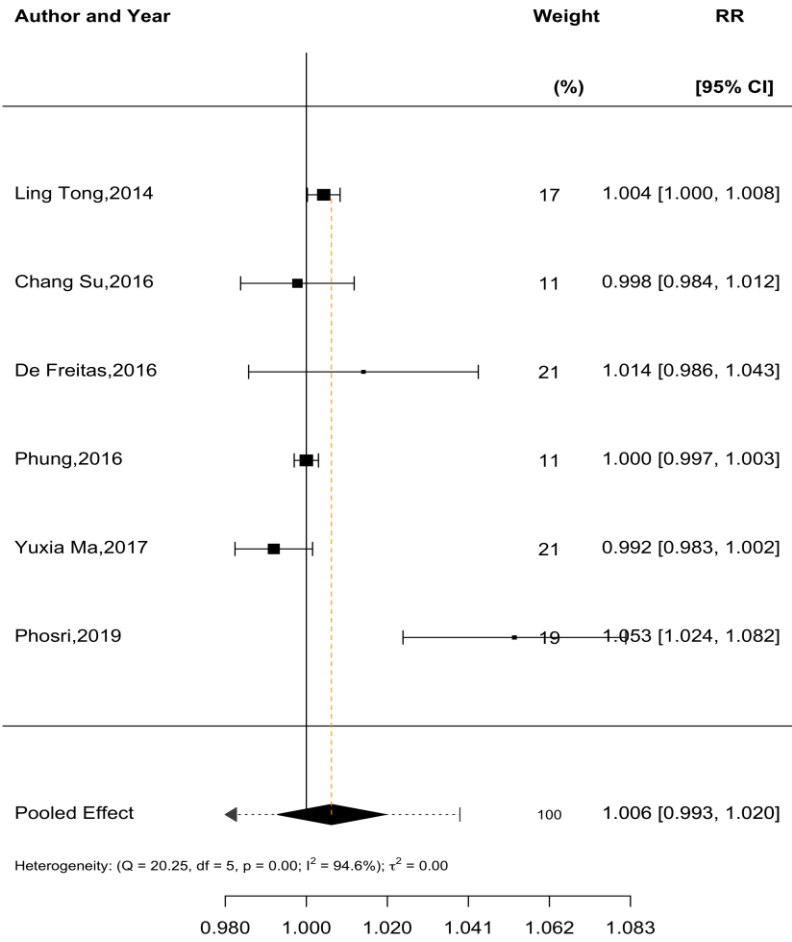

Supplement: Supplementary Figure A2. — Meta-analysis of SO2 exposure and cardiovascular disease morbidity. [file gh-20-1-1409-s3.pdf]
